# Supplementary material for: Identification of factors associated with morbidity and postoperative length of stay in surgically managed chronic subdural haematoma using electronic health records: a retrospective cohort study
Source: BMJ Open. 2020 Jun 30;10(6):e037385. doi: 10.1136/bmjopen-2020-037385 (PMC7328896; doi:10.1136/bmjopen-2020-037385)
Supplement: Supplementary data [file bmjopen-2020-037385supp001.pdf]

Supplementary Material: Impact of perioperative events extracted from an electronic health record on in-hospital length of stay after surgery for chronic subdural haematoma.

R-packages used in data manipulation and analysis

tidyverse[1]
mice[2]
naniar[3]
caret[4]
rlist[5]
plotROC[6]
ggsci[7]
survival[8]
survminer[9]
stargazer[10]

1 Wickham H. tidyverse: Easily Install and Load the 'Tidyverse'. R package version 1.2.1. https://cran.r-project.org/package=tidyverse%0A
2 Buuren S van, Groothuis-Oudshoorn K. mice: Multivariate Imputation by Chained Equations in R. 2019. doi:10.18637/jss.v045.i03
3 Tierney N, Cook D, McBain M, *et al.* naniar: Data Structures, Summaries, and Visualisation for Missing Data. R package version 0.4.2. 2019.https://cran.r-project.org/package=naniar
4 Kuhn M, et al. caret: Classification and Regression Training. R package version 6.0-84. https://cran.r-project.org/package=caret
5 Ren K. rlist: A Toolbox for Non-Tabular Data Manpiulation. R package version 0.4.6.1. 2016.
6 Sachs M. plotROC: A Tool for Plotting ROC Curves. Journal of Statistical Software. *J Stat Softw* 2017;**79**:1–19. doi:doi:10.18637/jss.v079.c02
7 Xiao N. ggsci: Scientific Journal and Sci-Fi Themed Color Palettes for 'ggplot2'. R package version 2.9. 2018.
8 T T. A package for Survival Analysis Version 2.38. 2015.
9 Kassambara Al. survminer: Drawing Survival Curves using 'ggplot2'. R package version 0.4.4.
10 Hlavac M. stargazer: Well-Formatted Regression and Summary Statistics Tables. R package version 5.2.1. 2018.

Definition of comorbidity

Comorbidity	WHO-ATC codes
**Anticoagulant/antiplatelet**	B01AC*, B01AA, B01AB, B01AD, B01AE, B01AF, B01AX
**Airways Disease**	R03*
**Heart Failure**	C07AB02, C07AB07, C07AG02, C07AB12, C03DA04 C03C*—C09*%
**History of CVS Disease**	C07AA, C07AB, C07AG01, C08C*, C08D*, C09DB, C09DX, C09BB C01DA, C01DX06, C08EX02

CVS = Cardiovascular, WHO-ATC = World Health Organisation Anatomical Therapeutic Classification code. Codes taken from the Rx-Risk score[65] * indicates that all drugs with codes below this level were counted as indicating this comorbidity. % for a diagnosis of heart failure based on drug codes C03C and C09 two drugs, one from each category must have been present to disentangle their use purely as anti-hypertensives. Anticoagulant use includes both antiplatelet and anticoagulants History of CVS disease was defined as those with angina or 'ischemic heart disease - hypertension' encoded in the Rx-Risk score.

Supplementary Table S1: *Patient comorbidities used in analysis and their calculation from a patient's prescribed medications, identified by World Health Organisation Anatomical Therapeutic Classification (WHO-ATC) code.*

Dictionary of WHO-ATC codes used in this analysis. Full details available at: https://www.whocc.no/atc_ddd_index/

A03FA01 = Metoclopramide
A04: Antiemetics and antinauseants

B01A: Antithrombotic agents
- B01AA = 'Vitamin K Antagonists'
- B01AB = Heparin Group
- **B01AC =** 'Platelet aggregation inhibitors excl. heparin'
- B01AD = Enzymes
- B01AE = Direct Thrombin Inhibitors
- B01AF = Direct Factor Xa inhibitors
- B01AX = Other antithrombotic agents

R03: Drugs for obstructive airway diseases

C07: Beta blocking agents
- *C07AB02 = Metoprolol*
- *C07AB07 = Bisoprolol*
- *C07AG02 = Carvedilol*
- *C07AB12 = Nebivolol*

C03C – C09:High-ceiling diuretics

C03DA04 = Eplerenone

C07AA = Beta blockers, non-selective
C07AB = Beta blockers, selective
C07AG01 = Labetalol
C08C = Calcium channel blockers with mainly vascular effects
C08D = Calcium channel blockers with direct cardiac effects
C09DB = Angiotensin II receptor blockers and calcium channel blockers
C09DX = Angiotension II receptor blockers, other combinations
C09BB = Ace inhibitors, calcium channel blockers
C01DA = Organic Nitrates
C01DX06 – Hexobendine
C08EX02 = perhexiline

J01 = Antibacterials for systemic use

N01BB01 = Bupivacaine
N02AA01 = Morphine
N02AB03 = Fentanyl

N05AD08 = Droperidol

R06AE03 = Cyclizine

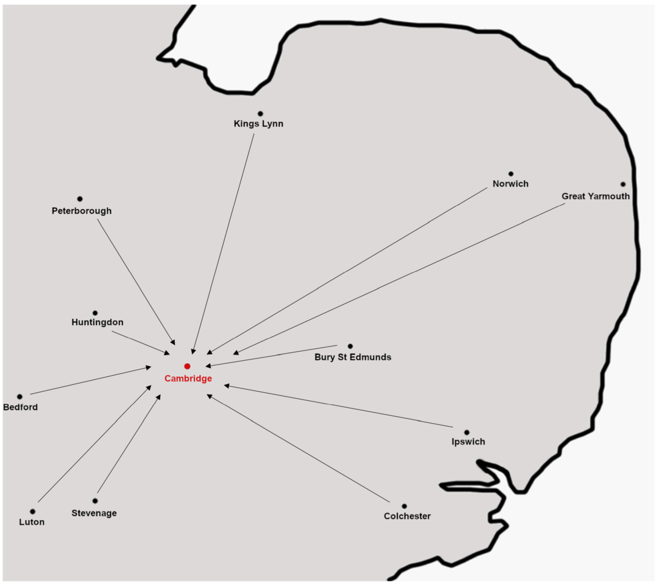

Referring hospitals are shown with black text, Cambridge neurosurgical centre shown in red. This only includes general referring hospitals although in reality, referrals are accepted from specialist hospitals such as Royal Papworth Hospital (Cardiac), and Broomfield Hospital (Plastics/Burns) that are not shown on this image.

Supplementary Figure S1: Referral region served by our neurosurgical centre.

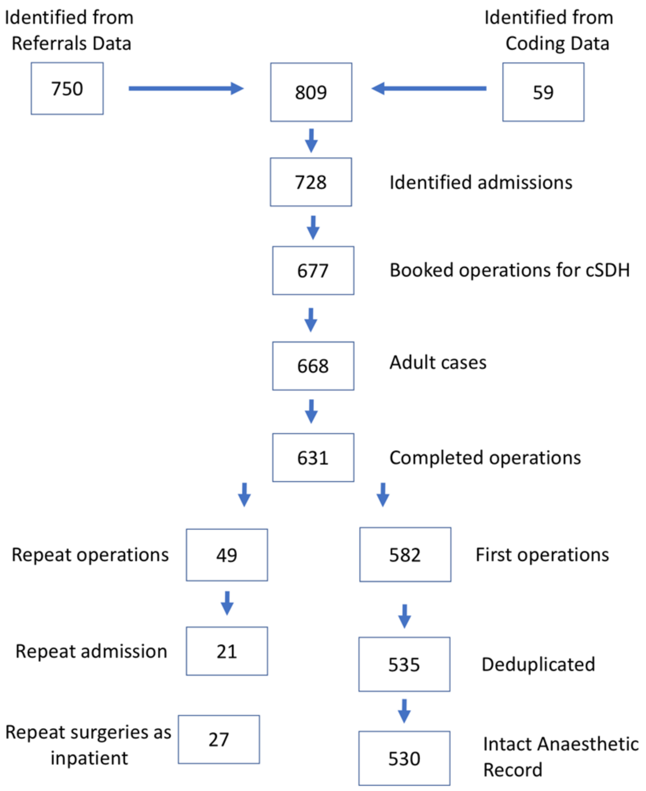

Supplementary Figure S2: **Identification of operated cases of chronic subdural haematoma at Cambridge University Hospitals NHS Foundation Trust (October 2014-January 2019).** CUH = Cambridge University Hospitals NHS Foundation Trust, cSDH = Chronic Subdural Haematoma. Cases from coding data are distinct individuals (from CUH) that were not found in the neurosurgical referrals database but were identified via diagnostic code. Discrepancy in number of admissions to number identified likely reflects cases that were eventually not transferred to CUH for surgery (for unknown reasons).

Missing Data patterns:

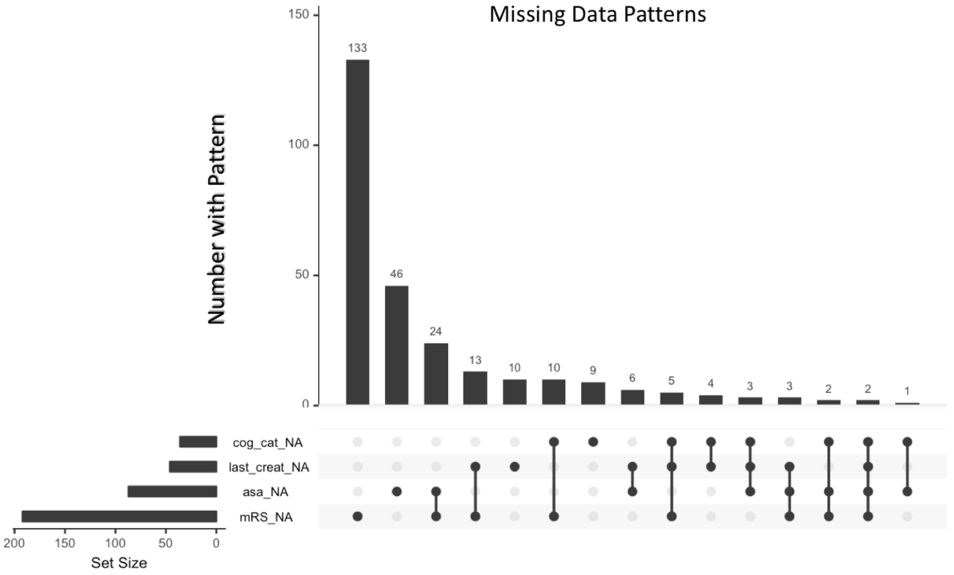

Supplementary Figure S3: Missing data patterns cog cat = presence of baseline cognitive impairment (Y/N), last creat = baseline creatinine measurement, asa = American Society of Anesthesiologists score, mRS = Modified Rankin Scale. Commonest pattern is that of missing mRS alone (n = 133). Vertical bars indicate number of individuals with each pattern (indicated by linked dots), horizontal bars indicate number of individuals with each missing variable.

Visualisation of Imputations

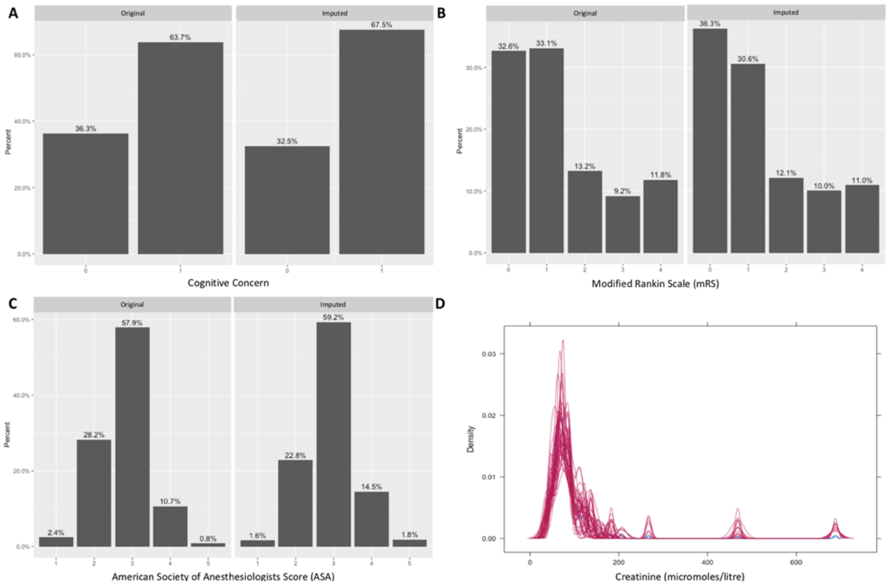

Supplementary Figure S4: *Visualisation of imputed values for A: Cognitive impairment documented on admission, B: Modified Rankin scale, C: American Society of Anesthesiologists (ASA) score, D: Creatinine.*

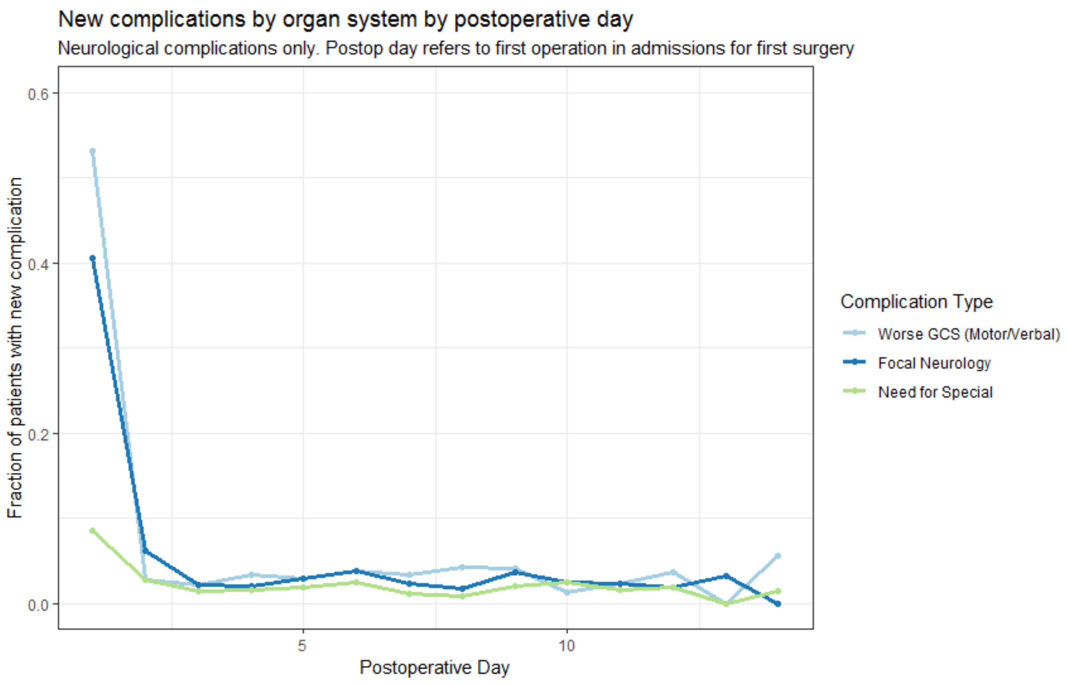

Supplementary Figure S5: Incident neurological complications by postoperative day.

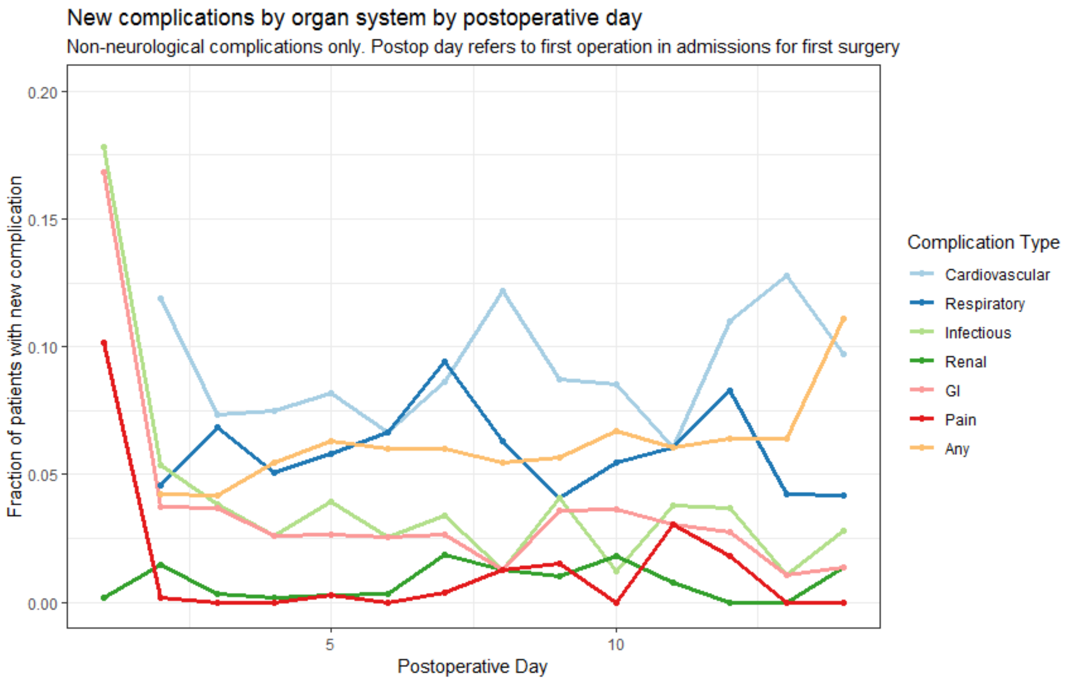

Supplementary Figure S6: Incident non-neurological complications by postoperative day.

	HR	2.5 %	97.5 %	*p*
**Demographics**				
**Age** *Per year*	0.993	0.987	1.000	0.042*
**Sex** *Male v Female*	1.204	0.998	1.453	0.053*
**Non-CUH patient**	2.197	1.541	3.131	<0.001*
**ASA 2** *All v ASA 1*	0.488	0.298	0.798	0.004*
**ASA 3**	0.338	0.208	0.547	<0.001*
**ASA4**	0.250	0.141	0.444	<0.001
**ASA5**	0.200	0.058	0.686	0.011*
**mRS 1** *All v mRS 0*	0.813	0.627	1.053	0.117
**mRS2**	0.837	0.593	1.182	0.312
**mRS3**	0.781	0.517	1.179	0.239
**mRS4**	0.670	0.456	0.985	0.041*
**Admission status**				
**GCS 15** *on admission versus any-other*	1.109	1.056	1.164	<0.001*
**Admission ePOMS** *per 1 domain increase*	0.915	0.865	0.967	0.002*
**Cognitive impairment** *Yes/No*	0.558	0.466	0.667	<0.001
**Comorbidities**				
**Airways disease** *Yes/No*	0.747	0.584	0.956	0.020*
**CVS disease** *Yes/No*	0.834	0.702	0.991	0.039*
**Heart Failure** *Yes/No*	0.698	0.560	0.870	0.001*
**Creatinine** *per umol/l increase*	0.958	0.922	0.995	0.028*
**Anticoagulated on admission** *Yes/No*	0.417	0.349	0.498	<0.001*
**Day of Surgery variables**				
**Pre-op deterioration** *(Yes/No)*	1.066	0.890	1.276	0.488
**Length of wait** *Per hour*	0.963	0.917	1.013	0.142
**Time MAP <80mmHg** *per 1 minute*	0.996	0.973	1.020	0.720
**Time *ETCO2* not 3-5kPa** *per 1 minute*	0.982	0.916	1.053	0.616
**Fentanyl dose** *per microgram increase*	1.012	0.988	1.037	0.330
**Volatile maintenance** *Yes/No*	0.996	0.838	1.183	0.964
**Operative time** *per 1 minute*	0.971	0.951	0.990	0.004*
**Postoperative**				
**Complications***per 1 domain increase in ePOMS*	0.574	0.521	0.632	<0.001*

Complete Case Analyses: Univariable analysis

Supplementary Table S2: Results from univariable cox regression analysis performed in complete cases for time to postoperative discharge after surgery for chronic subdural haematoma*. Hazard ratios (HR) of greater than 1 indicate an association with more rapid postoperative discharge. Asterisks indicate statistical significance at a p<0.1, threshold chosen for progression to multivariable modelling. Postoperative complications included as a time dependent covariate (by day). 95% CI = 95% Confidence interval. CUH = ASA = American Society of Anesthesiologists score, CUH = Cambridge University Hospitals NHS Foundation Trust, CVS = cardiovascular, ePOMS = electronic postoperative morbidity score, $ETCO_2$ = End tidal carbon dioxide measurement, GCS = Glasgow coma scale, MAP = mean arterial pressure, mmHg = millimetres of mercury, mcg = micrograms, mRS = modified Rankin scale.*

Complete Case Analysis: Multivariable analysis

	HR	95% CI	*p*-value
**Age** *(per year increase)*	0.99	0.98-1.01	0.301
**Sex** *(Male v Female)*	1.02	0.78-1.33	0.902
**GCS 15** *(v any-other value)*	1.03	0.93-1.15	0.581
**Referred patient**	2.40	0.69-8.30	0.167
**ASA** *(per 1 level increase)*	0.85	0.67-1.09	0.196
**Admission complications**	1.14	1.04-1.26	0.007*
**Airways disease** *(Yes/No)*	1.21	0.84-1.76	0.301
**CVS disease** *(Yes/No)*	1.13	0.84-1.52	0.424
**Anticoagulated on admission** *(Yes/No)*	0.52	0.40-0.69	<0.001*
**Heart failure** *(Yes/No)*	1.17	0.80-1.70	0.418
**mRS** *(per 1 level increase)*	1.03	0.93-1.13	0.566
**Baseline Creatinine** *(per 20umol/l increase)*	0.96	0.91-1.02	0.187
**Cognitive Concern** *(Yes/No)*	0.63	0.48-0.85	0.002*
**Operative time** (*per 10 minute increase)*	0.98	0.94-1.02	0.271
**Postoperative complications** *(per 1 domain increase in ePOMS)*	0.63	0.55-0.73	<0.001*

Supplementary Table S3: Results from multivariable cox regression model performed in complete cases (n = 260) for time to postoperative discharge after surgery for chronic subdural haematoma. *Hazard ratios (HR) of greater than 1 indicate association with a more rapid time to discharge. Asterisks indicate statistical significance at the 5% level. Postoperative complications included as a time dependent covariate (by day). 95% CI = 95% Confidence interval. CUH = ASA = American Society of Anesthesiologists score, CUH = Cambridge University Hospitals NHS Foundation Trust, CVS = cardiovascular, ePOMS = electronic postoperative morbidity score, GCS = Glasgow coma scale, mRS = modified Rankin scale.*

Sensitivity Analyses

	HR	95% CI	*p*-value
**Age** *(per year increase)*	0.99	0.99-1.00	0.169
**Sex** *(Male v Female)*	1.10	0.90-1.33	0.354
**Motor Score 6** *(v any-other value)*	0.97	0.83-1.12	0.674
**Referred patient**	1.53	1.01-2.29	0.042*
**ASA** *(per 1 level increase)*	0.88	0.75-1.04	0.135
**Admission complications**	1.05	0.99-1.12	0.102
**Airways disease** *(Yes/No)*	1.03	0.79-1.33	0.845
**CVS disease** *(Yes/No)*	0.96	0.78-1.19	0.715
**Anticoagulated on admission** *(Yes/No)*	0.45	0.37-0.54	<0.001*
**Heart failure** *(Yes/No)*	1.01	0.77-1.33	0.957
**mRS** *(per 1 level increase)*	0.99	0.91-1.08	0.835
**Baseline Creatinine** *(per 20umol/l increase)*	0.98	0.95-1.02	0.330
**Cognitive Concern** *(Yes/No)*	0.69	0.57-0.84	<0.001*
**Operative time** (*per 10 minute increase)*	0.99	0.97-1.01	0.374
**Postoperative complications** *(per 1 domain increase in ePOMS)*	0.61	0.55-0.68	<0.001*

Supplementary Table S4: Sensitivity analysis with total GCS replaced with an indicator variable for GCS motor score (6 or not) – highlighted in red.

	HR	95% CI	*p*-value
**Age** *(per year increase)*	0.99	0.99-1.00	0.046*
**Sex** *(Male v Female)*	1.15	0.94-1.39	0.168
**GCS 15** *(v any-other value)*	1.02	0.97-1.08	0.449
**Referred patient**	1.65	1.11-2.46	0.013*
**ASA** *(per 1 level increase)*	0.83	0.71-0.98	0.025*
**Admission complications**	1.01	0.95-1.07	0.841
**Airways disease** *(Yes/No)*	0.99	0.76-1.28	0.911
**CVS disease** *(Yes/No)*	0.96	0.78-1.19	0.732
**Anticoagulated on admission** *(Yes/No)*	0.45	0.37-0.54	<0.001*
**Heart failure** *(Yes/No)*	0.95	0.72-1.25	0.713
**mRS** *(per 1 level increase)*	0.97	0.89-1.07	0.561
**Baseline Creatinine** *(per 20umol/l increase)*	0.96	0.92-0.98	0.043*
**Cognitive Concern** *(Yes/No)*	0.65	0.53-0.79	<0.001*
**Operative time** (*per 10 minute increase)*	0.98	0.95-1.00	0.061

Supplementary Table S5: Sensitivity analysis, multivariable model with postoperative complication term excluded.
