# Supplementary material for: Identification of factors associated with morbidity and postoperative length of stay in surgically managed chronic subdural haematoma using electronic health records: a retrospective cohort study
Source: BMJ Open. 2020 Jun 30;10(6):e037385. doi: 10.1136/bmjopen-2020-037385 (PMC7328896; doi:10.1136/bmjopen-2020-037385)
Supplement: Author's manuscript [file bmjopen-2020-037385.draft_revisions.pdf]

BMJ Open is committed to open peer review. As part of this commitment we make the peer review history of every article we publish publicly available.

When an article is published we post the peer reviewers' comments and the authors' responses online. We also post the versions of the paper that were used during peer review. These are the versions that the peer review comments apply to.

The versions of the paper that follow are the versions that were submitted during the peer review process. They are not the versions of record or the final published versions. They should not be cited or distributed as the published version of this manuscript.

BMJ Open is an open access journal and the full, final, typeset and author-corrected version of record of the manuscript is available on our site with no access controls, subscription charges or pay-per-view fees (http://bmjopen.bmj.com).

If you have any questions on BMJ Open's open peer review process please email info.bmjopen@bmj.com

Utility of electronic health records in exploring associations between complex perioperative events morbidity and postoperative length of stay in surgically managed chronic subdural haematoma.

Journal:	*BMJ Open*
Manuscript ID	bmjopen-2020-037385
Article Type:	Original research
Date Submitted by the Author:	31-Jan-2020

Complete List of Authors:	Stubbs, Daniel; Cambridge University, University Division of Anaesthesia; Cambridge University, Healthcare Design Group<br>Davies, Benjamin; University of Cambridge, Department of Clinical Neurosurgery<br>Bashford, Tom; University of Cambridge , NIHR Global Health Research Group for Neurotrauma<br>Joannides, Alexis; Addenbrooke's Hospital, Department of Clinical Neurosciences; University of Cambridge, NIHR Global Health Research Group on Neurotrauma<br>Hutchinson, Peter; University of Cambridge, Academic Neurosurgery<br>Menon, David; NIHR Global Health Research Group for Neurotrauma, University of Cambridge<br>Burnstein, Rowan; Cambridge University Hospitals NHS Foundation Trust, Neurocritical Care Department and Department of Anaesthesia

Keywords:	ANAESTHETICS, EPIDEMIOLOGY, NEUROSURGERY, STATISTICS & RESEARCH METHODS, Quality in health care < HEALTH SERVICES ADMINISTRATION & MANAGEMENT

SCHOLARONE™
Manuscripts

I, the Submitting Author has the right to grant and does grant on behalf of all authors of the Work (as defined in the below author licence), an exclusive licence and/or a non-exclusive licence for contributions from authors who are: i) UK Crown employees; ii) where BMJ has agreed a CC-BY licence shall apply, and/or iii) in accordance with the terms applicable for US Federal Government officers or employees acting as part of their official duties; on a worldwide, perpetual, irrevocable, royalty-free basis to BMJ Publishing Group Ltd ("BMJ") its licensees and where the relevant Journal is co-owned by BMJ to the co-owners of the Journal, to publish the Work in this journal and any other BMJ products and to exploit all rights, as set out in our licence.

The Submitting Author accepts and understands that any supply made under these terms is made by BMJ to the Submitting Author unless you are acting as an employee on behalf of your employer or a postgraduate student of an affiliated institution which is paying any applicable article publishing charge ("APC") for Open Access articles. Where the Submitting Author wishes to make the Work available on an Open Access basis (and intends to pay the relevant APC), the terms of reuse of such Open Access shall be governed by a Creative Commons licence – details of these licences and which Creative Commons licence will apply to this Work are set out in our licence referred to above.

Other than as permitted in any relevant BMJ Author's Self Archiving Policies, I confirm this Work has not been accepted for publication elsewhere, is not being considered for publication elsewhere and does not duplicate material already published. I confirm all authors consent to publication of this Work and authorise the granting of this licence.

For peer review only

Utility of electronic health records in exploring associations between complex perioperative events morbidity and postoperative length of stay in surgically managed chronic subdural haematoma.

Stubbs DJ[1%], Davies B[2], Bashford T[3], Joannides A[4], Hutchinson PJ[5], Menon DK[6], Ercole A[7], Burnstein RM[7]

1: Wellcome Trust Clinical Research Fellow, Specialist Registrar in Anaesthesia, 3: Clinical Research Fellow NIHR Global Neurotrauma group, Specialist Registrar in Anaesthesia, 6: Professor, 7: Consultant in neuroanaesthesia and neurointensive care, University Division of Anaesthesia, Department of Medicine, Addenbrooke's Hospital, Hills Road, Cambridge, CB2 0QQ

2: Research fellow, Specialist registrar, 4: Clinical lecturer, 5: Professor, Division of Academic Neurosurgery, Department of Clinical Neurosciences, University of Cambridge, Addenbrooke's Hospital, Hills Road, Cambridge, CB2 0QQ

%: Corresponding author. Address for correspondence: djs225@cam.ac.uk

For peer review only

Word Count (Excluding Abstract): 3723

ABSTRACT

Introduction

Chronic subdural haematoma (cSDH) tends to occur in older patients, often with significant comorbidity. The incidence and effect of medical complications as well as the impact of the intraoperative management strategy are now attracting increasing interest.

Objectives:

We used electronic health record (EHR) data to study the profile of in-hospital morbidity and to examine associations between various intraoperative events and postoperative stay.

Design, setting and participants:

Single-centre, retrospective cohort of 530 cases of cSDH(2014-2019) surgically evacuated under general anaesthesia at a neurosciences centre in Cambridge, UK.

Methods and outcome definition:

Complications were defined using a modified electronic postoperative morbidity score. Association between complications and intraoperative care (time with mean arterial pressure (MAP) <80mmHg, time outside of end-tidal carbon dioxide ($ETCO_2$) range of 3-5.5kPa, maintenance anaesthetic, operative time, and opioid dose) on postoperative stay was assessed using Cox-regression.

Results:

53(10%) of patients suffered myocardial injury, 24(4.5%) suffered acute renal injury. On postoperative day 3 (D3) 280 (58% of remaining) inpatients suffered at least one complication. D7 rate was comparable (57%). Operative time was the only intraoperative event associated with postoperative stay (Hazard Ratio for discharge: 0.97[95% Confidence Interval 0.95-0.99]). Postoperative complications, were associated with longer postoperative stay (0.91[0.87-0.97] *per* complication). On multivariable analysis, complications (0.61[0.55-0.68]), anticoagulation (0.45[0.37-0.54]), and cognitive impairment (0.71[0.58-0.87]) were associated with time to discharge.

For peer review only

Conclusions

There is a high postoperative morbidity burden in this cohort which was associated with postoperative stay. We found no evidence of an association between intraoperative events and postoperative stay.

STRENGTHS AND LIMITATIONS OF THIS STUDY:

- First use of electronic health data to chart the time course of in-hospital complications in a neurosurgical cohort
- Use of multiple imputation to maximise power with clear comparison to complete case analysis for comparison
- Methodological approaches are scalable to other surgical and in-patient cohorts
- Main results limited by being a single centre, retrospective study and thus lack external validity

KEYWORDS.

- Anaesthetics
- Epidemiology
- Neurosurgery
- Statistics and Research methods
- Quality in health care

INTRODUCTION

Chronic subdural haematoma (cSDH) is a common neurosurgical pathology of encapsulated blood products beneath the dural membrane, driven by trauma and inflammation[1]. Although cSDH can be managed conservatively, surgical evacuation is favoured in the presence of neurological deficit. cSDH is predominantly a "disease of the elderly". It is also

For peer review only

For peer review only - http://bmjopen.bmj.com/site/about/guidelines.xhtml

associated with frailty and co-morbiditity [2–5]. Increased mortality at one year amongst individuals with a cSDH has led some to consider it a 'sentinel health event'[6]. In these respects there is a clear analogy with patients suffering a fracture of the neck of femur (NOF), a group whose outcomes have been recently improved by concerted efforts into the measurement, integration, and improvement of holistic perioperative care[7,8]. However, with cSDH, although a similarly effective surgical intervention exists, there has, as yet, been no demonstration of a systematic improvement in patient outcomes through analogous process changes.

To date, the impact of non-neurological morbidity in patients with cSDH has received little attention [9]. The reasons for this are likely multifactorial, but crucially there are significant challenges in adequately capturing patient morbidity using standard methodological approaches, which typically rely on cross-sectional assessment at set time points[10]. However, there is some evidence to suggest that non-neurological morbidity may be a significant problem. In a nationwide audit of UK cSDH management, 14% of 787 surgically treated patients suffered a complication, of which pneumonia was the most common[11]. The prevalence of such complications appears to increase with age [12,13]. The experience from parallel surgical fields would suggest [14] this morbidity has implications for both patient outcomes, and the delivery of cost-effective healthcare. This motivates a more detailed investigation into the relationship between perioperative morbidity and outcome in cSDH . An understanding of the impact of all stages of the perioperative pathway on patient outcome is vital if potential clinical and service benefits from tailored process change, analogous to those in the NOF population[8] are to be realised.

Surgery for cSDH is commonly performed under general anaesthesia (GA). Heterogeneity in anaesthetic practice has the potential to introduce variation in postoperative outcome, yet the impact of intraoperative events on outcome has received little attention in this field. Physiological changes, such as hypotension, can be a direct pharmacodynamic consequence of GA, the avoidance of which often requires pharmacological intervention. Importantly, even brief periods below a mean arterial pressure (MAP) of 80mmHg are known to be associated with end-organ damage[15] and it is reasonable to assume that the cSDH population may be particularly vulnerable. Furthermore, arterial carbon dioxide tension

For peer review only - http://bmjopen.bmj.com/site/about/guidelines.xhtml

($PaCO_2$) is no longer under homeostatic control during mechanical ventilation, which may have important implications for cerebral blood flow (CBF) and intracranial pressure (ICP)[16]. In cSDH, such changes in CBF correlate with clinical symptoms[17]. GA choice may also have postoperative effects. Opioids have numerous side effects that occur with increasing frequency in the elderly[18]. The varied pharmacodynamic effects of anaesthetic agents have the potential to differentially impact on both ICP and CBF[19], with intravenous anaesthesia associated with lower rates of certain complications such as nausea and vomiting[20]. However, a recent Cochrane review (of low quality evidence) failed to demonstrate a difference in delirium rate or length of stay between patients receiving a volatile or intravenous anaesthetic[21].

The advent of integrated electronic health records (EHR) gives the potential to capture complex events such as intraoperative physiology and to comprehensively measure in-patient morbidity across the entirety of an admission. New scoring systems are required to generate computable phenotypes from such complex, routinely collected data [22]. One such score, an electronic variant of the widely used postoperative morbidity score (POMS)[10], has equivalent discriminative performance in identifying discharge complexity or prolonged length of stay[22] (LOS). LOS is often critiqued as an outcome measure in improvement effort due to the impact of non-clinical events[23]. The complex relationship between clinical and non-clinical events which conspire to impact length of stay is likely to vary between patients with differing disease processes. Regardless, its utility from the perspective of impact on hospital bed occupancy in both specialist and non-specialist centres is inarguable; with mounting health service pressures bed utilisation and patient throughput with minimal accrued morbidity is of crucial importance.

The aim of this study was to use routinely collected EHR data to examine, for the first time, the impact of intraoperative events and medical complications on postoperative length of neurosurgical centre stay in patients having surgery for cSDH.

METHODS

For peer review only

Cohort Formation

This is a single centre, retrospective evaluation of surgically treated cSDH at Cambridge University Hospitals NHS Foundation Trust (CUH) between 26th October 2014 and 5th January 2019. CUH offers neurosurgical and trauma services to a population of approximately 5.8 million in the East of England (**Supplementary Figure S1)** [24]. 809 cases of cSDH were identified from a neurosurgical referrals database and electronic operation log, coded contemporaneously by neurosurgeons. After excluding missing records, duplicates, and incorrect procedures, 530 patients were included in this retrospective cohort (**Supplementary Figure S2**). The majority of patients (491, 92.5%) were transferred from other hospitals (*n = 14*). Anonymised data for each case was extracted from our EHR (Epic Systems Corporation, Verona, WI) by our clinical informatics team. The study forms part of a local service improvement programme, with local service evaluation approval obtained in line with UK procedure (ref: PRN 7703).

Patient and Public Involvement Statement:

Due to the retrospective and evaluative nature of the project no formal patient and public involvement work was undertaken.

Covariate definitions

Perioperative medical complications

We utilised a variant of the electronic postoperative morbidity score (ePOMS)[22] to determine the presence of morbidity across 9 distinct domains, following key organ systems (respiratory, cardiovascular, neurological, renal, haematological, gastro-intestinal, infectious, reoperation/wound, and pain) (**Table 1)**. To capture a greater number of dimensions of neurological morbidity, we further adapted the score to include deterioration in motor or verbal components of the Glasgow coma scale (GCS) (from admission) and the presence of asymmetric motor power. Therefore, the total score for an individual on any given day ranged between 0 and 11. Electronic observation, drug, and laboratory result charts were extracted from the EHR and the daily ePOMS generated for each patient.

For peer review only

Physiological criteria (e.g. heart rate) were fulfilled if 2 or more instances occurred on a given day compared to a single event for investigation and drug-based criteria.

Domain	Diagnostic Criteria	Notes
**Respiratory**	Need for supplementary oxygen	
**Cardiovascular**	HR > 100<br>SBP < 100<br>Positive Troponin Test	
**Neurological**	Need for 1:1 nursing observation<br>Motor/verbal score worse than referral<br>Focal neurology	Surrogate for hyperactive delirium<br>Documented mismatch between left/right sided arm/leg strength at any stage
**Renal**	Rise in creatinine to $\geqslant$ 1.5x baseline	Last recorded creatinine prior to surgery
**GI**	Administered anti-emetic	Defined by the following WHO ATC codes: a04**, a03fa01, r06ae03, n05ad08
**Pain**	Need for IV opioids or local anaesthetic infusion on a given day	Drugs identified by following WHO ATC codes: n02aa01, n02ab03, n01bb01
**Recurrence**[%]	Reoperation	EPOMS originally identifies severe wound infection by need for further surgery. In this context reoperation for the same procedure is being used to identify re-accumulation of cSDH
**Infection**	Temperature $\geqslant$ 38°C<br>Receiving Antibiotics on a given day	Antibiotics defined by following WHO ATC codes: j01**

For peer review only

**Haematological**	Transfused with blood product	Including red cells, platelets, FFP, cryoprecipitate.

Table 1: Definitions of the adapted electronic postoperative morbidity score (EPOMS) used for daily assessment of complications in a retrospective cohort of operated chronic subdural haematoma. HR = Heart rate, SBP = Systolic blood pressure, GCS = Glasgow Coma Scale, GI = Gastrointestinal, WHO ATC = World Health Organisation Anatomical therapeutic chemical classification, IV = intravenous, FFP = fresh frozen plasma. If multiple potential criteria are listed then an individual is positive in that domain if any of these are met. % In the original EPOMS score this would correspond to the wound category. * indicates additional criterion included in this variant of EPOMS from those previously published. ** indicates that all drugs below this level of ATC code were included. A dictionary of relevant ATC codes is available in the supplementary material.

For peer review only

Generation of intra-operative covariates

Automatically recorded physiological measurements (blood pressure, heart rate) and inspired/expired gas measurements were extracted from our EHR at minute resolution throughout GA. Blood pressure was measured both non-invasively (typically at a frequency of more than every 5 minutes[25]) and, for a subset of patients, invasively from an arterial catheter. All available MAP values were combined and linearly interpolated. Total time (in minutes) spent below a threshold of 80mmHg[15] was calculated. In an effort to reduce artefacts, values of 0 or >200mmHg were excluded. Maintenance anaesthetic was deemed inhalational if there were non-zero values of an inhalational anaesthetic agent. Text labels of manually recorded drug administrations were automatically converted using a lookup table matching drugs to their corresponding World Health Organisation anatomical therapeutic classification (WHO-ATC) code[26]. All administrations of a drug with a code of N02A (opioid drug class) were extracted along with their dose. All opioid administrations were expressed as fentanyl equivalents using published conversions[27] as this is the most commonly used opioid in our neuroanaesthetic practice. Brain injury guidelines suggest that ventilation is adjusted to keep the level of carbon dioxide measured in arterial blood ($PaCO_2$) less than 6 and above 3.5kPa[28]. As end tidal values underestimate the true arterial gas tension by between 0.5-1kPa, time above an $ETCO_2$ of 5kPa and time below an $ETCO_2$ of 3kPa were both counted. Both time periods were added to give a total time outside of CO_2 range.

Other Covariates

To control for residual confounding in any observed associations we also extracted key admission characteristics from the EHR and referral database. These included: age, sex, American society of Anesthesiologists (ASA) score, modified Rankin scale (mRS), admission location (direct or from a referring hospital), baseline neurological status (GCS 15 yes/no, motor score 6 yes/no), admission creatinine, and key comorbidities. We also controlled for any preoperative deterioration (a worsening between ePOMS score on day of admission and day of surgery) as well as the length of wait for surgery. As the majority of patients referred for surgery had no previous admission on record the baseline problem list of comorbidities was frequently incomplete. Comorbidities were inferred from admission medications using the 'Rx-Risk' score which uses WHO-ATC codes to determine the presence of specific comorbidities[29] rather than using discharge clinical coding to avoid potential reverse causation (e.g. where an in-hospital myocardial infarction (MI) was wrongly interpreted as a pre-existing history of myocardial infarction). Preadmission anticoagulant/antiplatelet use, airways disease, heart failure, or cardiovascular disease were determined using medication records reconciled in the first 24 hours of an individual's admission. Full codes used to determine comorbidity are shown in **supplementary information.**

Statistical techniques

All analysis was performed in R (v 3.5.3)[30]. Effect of covariates on postoperative length of stay was performed using Cox regression. ePOMS scores were included as a time dependent covariate calculated per day, all other variables were taken to exert effect over the entire postoperative period. Univariable analysis was performed for all variables, those with a $p<0.1$ on initial regression were carried forward for multivariable modelling. At this stage statistical significance was taken as $p<0.05$. All analyses were conducted in both complete case and multiple imputed datasets.

<u>Handling of missing data</u>

Daily entries in the EHR for calculating ePOMS scores (such as blood results, observations, and administered drugs) were assumed to be accurate (e.g. if no troponin test was ordered on that day we did not impute a value). Anaesthetic chart data (observations,

For peer review only - http://bmjopen.bmj.com/site/about/guidelines.xhtml

measurements of gas tension, and administered drug charts) were also taken to be accurate due to automated data capture. Baseline patient variables (such as ASA score) were imputed using multiple imputation with chained equations (MICE)[31]. This was performed using the R packages 'mice'[32], and 'naniar'[33]. A full list of R packages used is available in the **supplementary material.** A multi-level imputation model, clustered by individual, was used to allow imputation of baseline data alongside longitudinal ePOMS scores[34]. Visualisation of missing data patterns are shown in **Supplementary Figure S3.** Four variables had missing data; baseline creatinine (missing in 46 – 8.7%), cognitive status (36 – 6.8%), ASA score (87 – 16.1%), and mRS (192 – 36.2%). All four were missing in only 2 cases. 40 imputed datasets were formed. Comparison of imputed and observed values can be seen graphically in the **Supplementary Figure S4**. All results presented in the results reflect pooled analyses across all imputed datasets, corresponding complete case analyses are in the **supplementary material.**

RESULTS

Inpatient morbidity

Over the study period, 13 individuals (2.4%) died during admission. 49 (9.2%) of patients were re-operated for their cSDH, 28 during their index admission. 53 (10%) of individuals exhibited some degree of postoperative troponin rise, with 24 (4.5%) developing acute kidney injury. 346(65.2%) patients had a degree of preoperative deterioration in their medical state between admission and surgery as determined by an increase in their ePOMS between these timepoints.

The prevalence of each complication subtype shown by postoperative day (between the day of surgery and postop day 14) is demonstrated in **Figures 1 and 2**. There was an obvious restoration in focal neurology over the first two postoperative days **(Figure 1),** although such a trend was not apparent in those needing 1:1 observation at the bedside.

The apparently high prevalence of respiratory 'complications' on day of surgery and postop day 1 is likely to be spurious, with an ePOMS event triggered instead by the routine use of supplemental oxygen to counteract post-anaesthetic effects or for the treatment of postoperative pneumocephalus. Prevalence of infective complications

For peer review only

appeared the highest between days 3 and 10 (**Figure 2**). By day 3, 280 individuals (58% of 484 remaining inpatients) had at least one complication, a comparable rate to day 7 - 138 of 242 remaining inpatients (57%).

**Variable**	**Median [IQR]**
**Age** *years*	77 [69-84]
**Creatinine** *μmol/l*	73 [61-89]
	n **(%)**
**Male**	376 (70.8)
**ASA ≥ 3**	271 (61.0)*
**Cognitively Impaired**	270 (54.5)*
**Admission GCS 15**	342 (64.4)
**Admission Motor Score 6**	490 (92.3)
**mRS ≥ 2**	105 (31.0)*
**Anticoagulants/Antiplatelets**	233 (43.9)
**CVS disease**	239 (45.0)
**Heart failure**	101 (19.0)
**Airways disease**	75 (14.1)

Table 1: Baseline characteristics of a retrospective cohort of operated chronic subdural haematomas at Cambridge University Hospitals NHS Foundation Trust (*n=530*). ASA = American Society of Anesthesiologists score, GCS = Glasgow Coma Scale, mRS = Modified Rankin Score, CVS = Cardiovascular, Motor Score refers to score on the motor (movement) component of the Glasgow Coma Scale. ASA of 3 or more indicates presence of significant systemic comorbidity, modified Rankin scale of 2 or more indicates a level of disability that impedes normal activity * indicates that value is calculated only on those with recorded values.

Length of Stay

Overall median length of stay in our centre was 6.7[IQR:4.2-10.6] days. For local (directly admitted) patients this was 8.9 [IQR:5.2-16.3] days versus 6.5 [IQR:4.1-10.1] days for those referred from elsewhere. Postoperative length of stay was 4.9[3.0-8.8]

For peer review only

days in referred patients, compared to 7.9[3.4-15.5] days in local patients. Time to surgery (after admission to our centre) was similar in both groups (0.8[0.2-1.4] days for local patients *v* 0.8[0.4-1.8] days for referred patients). Postoperative time to discharge, dichotomised by referral source is shown in **Figure 3.**

Association of intraoperative events and complications on postoperative length of stay.
From the examined operative variables only operative time demonstrated a significant association (HR 0.97 [0.95-0.99]) on univariable analysis. Postoperative complications were associated with prolonged postoperative stay (HR 0.57 [0.52-0.63]) when included as a time dependent covariate. Baseline variables including age, sex, ASA score, mRS, medical state, and comorbidities were associated with longer time to postoperative discharge (**Table 2)** whilst a favourable GCS and being from a referred hospital was associated with a more rapid discharge from our neurosciences centre.

18 of these variables were taken forward to multivariable modelling (**Table 3**). This was done to ensure maximal control for confounding. On multivariable analysis, postoperative complications (HR 0.61 [0.55-0.68]), cognitive concerns (HR 0.71 [0.58-0.87]), and anticoagulation (HR 0.45 [0.37-0.54]) were all associated with longer time to discharge. Admission GCS (HR 1.03 [0.97-1.08]) and referral source (HR 1.46 [0.98-2.17]) were no longer significant in the final model. On complete case analysis the pattern was similar except for an identified association with more rapid discharge with increase admission complication (ePOMS) score (HR complete case 1.14 [1.04-1.26] *vs* HR MI 1.06 [0.99-1.12]) (**Supplementary Table S3**).
Based on our results, two sensitivity analyses were performed to attempt to understand the apparent lack of association between admission GCS and postoperative stay. Firstly, total GCS was replaced with an indicator for motor score (6 or not) (**Supplementary Table S4**) and secondly any potential mediating effect of postoperative complications was examined by removing this covariate (**Supplementary Table S5)**. In the first analysis, motor score was similarly not associated with postoperative time to discharge. When exploring whether complications were a potential mediator, no significant association with GCS was revealed in the subsequent model, although significant associations with admission ASA score, renal function, and age did become apparent (**Supplementary Table S5**).

For peer review only - http://bmjopen.bmj.com/site/about/guidelines.xhtml

	HR	95% CI	*p*-value
**Demographics**			
**Age** *(per year increase)*	0.99	0.99 – 0.99	*0.042**
**Sex** (*male v female)*	1.20	0.99-1.45	*0.053**
**Referred patient**	2.20	1.54-3.13	*<0.001**
**ASA 2** (*All v ASA 1)*	0.50	0.31-0.82	*0.006**
**ASA 3**	0.34	0.21-0.55	*<0.001**
**ASA 4**	0.26	0.15-0.45	*<0.001**
**ASA 5**	0.22	0.07-0.68	*0.009**
**mRS 1** *(All v mRS 0)*	0.86	0.67-1.11	*0.249*
**mRS 2**	0.83	0.58-1.18	*0.299*
**mRS 3**	0.73	0.47-1.11	*0.142*
**mRS 4**	0.68	0.46-0.98	*0.040**
**Admission Status**			
**GCS 15 on admission** (*v any-other)*	1.11	1.06-1.16	*<0.001**
**Admission ePOMS** *(per 1 domain increase*)	0.91	0.87-0.97	*0.002**
**Cognitive impairment** (*yes/no*)	0.57	0.48-0.69	*<0.001**
**Comorbidities**			
**Airways disease**	0.75	0.58-0.96	*0.020**
**CVS disease**	0.83	0.70-0.99	*0.040**
**Heart Failure**	0.70	0.56-0.87	*0.001**
**Anticoagulated on admission**	0.42	0.35-0.50	*<0.001**
**Creatinine** (*per 20umol/l increase*)	0.96	0.92-0.99	*0.022**
**Day of Surgery**			
**Preop deterioration** *(Yes/No)*	1.07	0.89-1.28	*0.488*
**Length of wait** *(per hour)*	0.96	0.92-1.01	*0.142*

For peer review only - http://bmjopen.bmj.com/site/about/guidelines.xhtml

**Time MAP <80mmHg** *(per 10 minute increase)*	0.99	0.97-1.02	*0.720*
**Time $ETCO_2$ not 3-5kPa** *(per 10 minute increase)*	0.98	0.92-1.05	*0.616*
**Fentanyl dose** *(per 25 mcg increase)*	1.01	0.99-1.04	*0.330*
**Volatile maintenance**	0.99	0.84-1.18	*0.964*
**Operative time** *(per 10 minute increase)*	0.97	0.95-0.99	*0.004**
**Postoperative**			
**Complications** *(per 1 domain increase in ePOMS)*	0.57	0.52-0.63	*<0.001**

Table 2: Results from univariable cox regression of effects of specific variables on postoperative length of stay in a cohort of patients undergoing surgery for cSDH (*n =530)* pooled across *m=40* imputed datasets. Hazard ratios (HR) of more than 1 indicate a more rapid time to postoperative discharge. Asterisks indicate statistical significance at *p<0.1*, chosen threshold for inclusion in subsequent multivariable modelling. Postoperative complications included as a time dependent covariate (by day). 95% CI = 95% Confidence interval. ASA = American Society of Anesthesiologists score, CVS = cardiovascular, ePOMS = electronic postoperative morbidity score, $ETCO_2$ = End tidal carbon dioxide measurement, GCS = Glasgow coma scale, MAP = mean arterial pressure, mmHg = millimetres of mercury, mcg = micrograms, mRS = modified Rankin scale.

Multivariable analysis

	**HR**	**95% CI**	***p*-value**
**Age** *(per year increase)*	0.99	0.99-1.00	0.114
**Sex** *(Male v Female)*	1.09	0.90-1.33	0.351
**GCS 15** *(v any-other value)*	1.03	0.97-1.08	0.377
**Referred patient**	1.46	0.98-2.17	0.063
**ASA** *(per 1 level increase)*	0.90	0.77-1.06	0.217
**Admission complications**	1.06	0.99-1.12	0.093
**Airways disease** *(Yes/No)*	1.03	0.79-1.34	0.819
**CVS disease** *(Yes/No)*	0.96	0.77-1.18	0.676

For peer review only - http://bmjopen.bmj.com/site/about/guidelines.xhtml

**Anticoagulated on admission** *(Yes/No)*	0.45	0.37-0.54	<0.001*
**Heart failure** *(Yes/No)*	1.02	0.77-1.33	0.908
**mRS** *(per 1 level increase)*	0.99	0.91-1.09	0.885
**Baseline Creatinine** *(per 20umol/l increase)*	0.98	0.94-1.01	0.242
**Cognitive Concern** *(Yes/No)*	0.71	0.58-0.87	<0.001*
**Operative time** *(per 10 minute increase)*	0.99	0.91-1.01	0.357
**Postoperative complications** *(per 1 domain increase in ePOMS)*	0.61	0.55-0.68	<0.001*

Table 3: Multivariable Cox regression model for postoperative time to discharge in a cohort of patients undergoing surgery for chronic subdural haematoma (*n* = 531), pooled across *m=40* imputed datasets. Hazard ratios (HR) of greater than 1 indicate association with a more rapid time to discharge. Asterisks indicate statistical significance at the 5% level. Postoperative complications included as a time dependent covariate (by day). 95% CI = 95% Confidence interval. ASA = American Society of Anesthesiologists score, CVS = cardiovascular, ePOMS = electronic postoperative morbidity score, $ETCO_2$ = End tidal carbon dioxide measurement, GCS = Glasgow coma scale, mRS = modified Rankin scale.

DISCUSSION

To our knowledge, this is the first study, in cSDH, to use a comprehensive evaluation of an EHR to detect postoperative morbidity, evaluate clinical interventions, such as anaesthetic events, and explore their impact on LOS.

We highlight a significant degree of post-operative morbidity in patients treated surgically for cSDH. Despite comparable cSDH recurrence rates of 9.2% (9% in a UK wide audit of practice[11]) in-patient morbidity captured by the ePOMS demonstrated a prevalence of medical complications in over 50% of patients in the two weeks after surgery. 13% of patients demonstrated a rise in serum troponin or an episode of renal injury after surgery. This morbidity was significantly associated with longer stay in the neurosurgical centre, raising the question of whether efforts to pre-empt or aggressively manage such morbidity could afford more efficient delivery of healthcare and, importantly, improved patient outcome. The total in-hospital burden of morbidity is likely higher than our estimate, as the single-centre nature of our evaluation precludes the measurement of complications

For peer review only - http://bmjopen.bmj.com/site/about/guidelines.xhtml

occurring in a patient's referring hospital both before referral, and after discharge from, the neuroscience centre.

The restriction of data to a single, tertiary centre, in which the majority of patients were transferred from other hospitals for treatment, precludes associations with total inpatient stay as unmeasured events, including bed and transport availability, will also be important. An idea of this can be gained by considering the difference in patient stay between local and referred patients in our study **(Figure 1)** although this assumes that local and referred patient cohorts are otherwise comparable in their characteristics and management. Such assumptions can only be examined in the context of further work. Regardless, our presented findings are likely to be highly relevant to tertiary neurosurgical centres.

Cognitive function on admission, previous anticoagulation, and postoperative complications were all associated with longer time to discharge. Importantly, admission GCS was not associated with postoperative stay. This requires consideration; one interpretation could be that any impact of neurology on postoperative course may be mediated through the included postoperative complication term (e.g. low GCS is associated with length of stay through a predisposition to complications like pneumonia). On a sensitivity analysis there was still no association after removal of the complication term. A larger question is to consider which dimension of neurological morbidity is best for prognostication in this patient cohort, both in terms of underlying pathophysiology and data coding. For instance, an individual with a hemiparesis arising from a non-dominant hemisphere lesion has an obvious neurological deficit but would likely be recorded as possessing a motor score of 6 and a GCS of 15 thus weakening any observed association. Unpicking these issues and the co-linearity inherent within these overlapping variables is difficult and should be explored in larger data sets.

We have attempted to control for many variables identified in previous work in traumatic neurosurgical patients[35]. The significant change in the observed HR for complications between univariable and multivariable analysis (0.91 *v* 0.61) suggests we have controlled for significant negative confounders in our final model. The shift in HR for referral source on multivariable modelling suggests that at least some of its observed association with time to discharge reflects confounding or mediation by included variables, perhaps reflecting

For peer review only

differences in patient cohorts or management (such as types or degree of complications preventing repatriation) between local and referred patients. These findings require further evaluation in future studies and distinct cohorts.

Although not widely examined, studies that have considered medical complications in cSDH have suggested they may be a significant burden. For instance, in a 2014 cross-sectional survey of UK neurosurgical centres, 14% of 787 surgically treated cSDH were found to have suffered a complication. In this survey morbidity was defined as, '…any adverse event occurring during inpatient stay…' [36], with a predefined audit target of <10%. In our study, ePOMS defined morbidity peaked at 65.2%. This higher prevalence likely reflects the sensitivity of the scoring system. One interpretation would be that we are measuring 'lower-level' morbidity that has not been captured in previous studies. However, we interpret the consistent association of such ePOMS defined morbidity with LOS as indicating the clinical relevance of such events. As EHR become more widespread the utilisation of phenotyping techniques such as the ePOMS will become increasingly important but, in the short term, create a degree of difficulty in allowing direct comparisons to previously published literature.

The implementation of electronic health records across the health service (NHS) is a government priority[37] in recognition of their numerous advantages . The evidence from our study is that interrogation of the EHR is a valuable addition to the improvement process, allowing the extraction and analysis of complex events (such as minute-by-minute physiological measurements) and computable phenotypes. This approach enables the evaluation of the impact of previously unmeasured clinical interactions on patient outcomes at different stages of the patient-journey. The importance of integrated care at a system level is widely recognised in the care of traumatic brain injury. Here, interventions and further research can be prioritised based on an understanding of the drivers of outcome in each phase of care[38]. The nature of the cSDH referral pathway and the inherent complexity of the patient cohort means simple interventions to improve outcome are unlikely to be successful and a similar 'system' wide approach may be an appropriate response. Studies such as ours which examine hitherto unexplored events, will be essential for defining both the scope of the problem and areas for improvement.

For peer review only - http://bmjopen.bmj.com/site/about/guidelines.xhtml

In this study we have evaluated the impact of potentially significant elements of intra-operative care for the first time in a cohort of cSDH patients using our EHR; Significant associations with LOS were not found. We defined our covariates based on time outside of ranges associated with adverse outcome in other surgical specialities[15] or recommended as physiologically appropriate targets in more severe head injury[28,39]. Periods of time (10 minutes or more) spent at a MAP of <80mmHg are associated with end-organ complications in non-cardiac surgery[15]. In the same setting, a 'dosing effect' has also been suggested, with shorter periods of time under lower MAP values also associated with complications. 80mmHg was chosen for this project due to its recommendation in other settings of neurosurgical practice [39] but perhaps total time below this threshold, without considering a dosing effect may explain the lack of any observed association. Similarly, it may be that MAP targets should be individualised based on patient characteristics such as comorbidity and exploring such interactions should be an active area of future research.

The EHR does have limitations, the majority of which are grounded in the availability of readily extractable data encapsulating key clinical characteristics such as radiological diagnoses (e.g. impact on midline shift) or the specific events which may occur during an operative procedure. The absence of related information such as comorbidities required the use of novel methods to infer this phenotype[29]. Although this methodology has been used in a large Australian database, the score has not, to our knowledge, been used in the surgical setting before this. The need for such methods may vary by cohort. We hypothesise that for individuals admitted electively or who have had previous treatment in our centre that comorbidity documentation will be more complete. Hospital coding for SDH similarly does not confidently delineate acute from chronic SDH. [40] In order to maintain anonymity of individual cases, this could not be corroborated by extracting radiographic or operation details. Instead in this study, this was mitigated through linkage with the Neurosurgical referral database, where cases are contemporaneously coded by a Neurosurgical clinician. Cases identified through coding data were refined by excluding those with associated major traumatic injuries to minimise the inclusion of cases of acute SDH. It is however possible that some cases were incorrectly included.

For peer review only - http://bmjopen.bmj.com/site/about/guidelines.xhtml

Conclusion

This study provides the first comprehensive evaluation of inpatient care for surgically treated cSDH using an integrated EHR. Perioperative morbidity was frequent and associated with increased postoperative LOS. Preoperative anticoagulation, admission GCS, cognitive dysfunction, and referral source were also associated with postoperative length of stay. This study identifies key areas to explore in quality improvement initiatives, whilst providing a methodological framework relevant to Neurosurgery, as well as other surgical and inpatient specialities.

For peer review only

References:

1 Edlmann E, Giorgi-Coll S, Whitfield PC, *et al.* Pathophysiology of chronic subdural haematoma: Inflammation, angiogenesis and implications for pharmacotherapy. *J Neuroinflammation* 2017;**14**:1–13. doi:10.1186/s12974-017-0881-y
2 Fogelholm R, Waltimo O. Epidemiology of chronic subdural haematoma. *Acta Neurochir (Wien)* 1975;**32**:247–50.
3 Alexander E, Wagner E, Buchner D, *et al.* Do surgical brain lesions present as isolated dementia? A population- based study. *J Am Geriatr Soc* 1995;**43**:138–43.
4 Kudo H, Kuwamura K, Izawa I, *et al.* Chronic subdural hematoma in elderly people: present status on Awaji Island and epidemiological prospect. *Neurol Med Chir (Tokyo)* 1992;**32**:207–9.
5 Bartek J. J, Sjavik K, Stahl F, *et al.* Surgery for chronic subdural hematoma in nonagenarians: A Scandinavian population-based multicenter study. *Acta Neurol Scand* 2017;**136**:516–20.
6 Dumont TM, Rughani AI, Goeckes T, *et al.* Chronic subdural hematoma: A sentinel health event. *World Neurosurg* 2013;**80**:889–92.
7 Royal College of Physicians. *National Hip Fracture Database (NHFD) annual report 2017*. London: : Royal College of Physicians 2017. doi:ISBN978-1-86016-577-1
8 Gilchrist N, Dalzell K, Pearson S, *et al.* Enhanced hip fracture management: Use of statistical methods and dataset to evaluate a fractured neck of femur fast track pathway—pilot study. *N Z Med J* 2017;**130**:91–101.
9 Chari A, Hocking KC, Broughton E, *et al.* Core Outcomes and Common Data Elements in Chronic Subdural Hematoma: A Systematic Review of the Literature Focusing on Reported Outcomes. *J Neurotrauma* 2016;**33**:1212–9. doi:10.1089/neu.2015.3983
10 Grocott MPW, Browne JP, Van der Meulen J, *et al.* The Postoperative Morbidity Survey was validated and used to describe morbidity after major surgery. *J Clin Epidemiol* 2007;**60**:919–28. doi:10.1016/j.jclinepi.2006.12.003
11 Brennan PM, Kolias AG, Joannides AJ, *et al.* The management and outcome for patients with chronic subdural hematoma: a prospective, multicenter, observational cohort study in the United Kingdom. *J Neurosurg* 2016;**127**:732–9. doi:10.3171/2016.8.jns16134

For peer review only - http://bmjopen.bmj.com/site/about/guidelines.xhtml

12 Adhiyaman V, Chattopadhyay I, Irshad F, *et al.* Increasing incidence of chronic subdural haematoma in the elderly. *QJM-AN Int J Med* 2017;**110**:375–8. doi:10.1093/qjmed/hcw231
13 Munoz-Bendix C, Pannewitz R, Remmel D, *et al.* Outcome following surgical treatment of chronic subdural hematoma in the oldest-old population. *Neurosurg Rev* 2017;**40**:461–8. doi:10.1007/s10143-016-0803-y
14 National Confidential Enquiry into Perioperative Outcome & Death. An age old problem. 2010. doi:10.1136/bmj.39356.408102.59
15 Wesselink EM, Kappen TH, Torn HM, *et al.* Intraoperative hypotension and the risk of postoperative adverse outcomes: a systematic review. *Br J Anaesth* 2018;**121**:706–21. doi:10.1016/j.bja.2018.04.036
16 Tameemm A, Krovvidi H. Cerebral physiology. *Contin Educ Anaesthesia, Crit Care Pain* 2013;**13**:113–8. doi:10.1093/bjaceaccp/mkt001
17 S. I, T. K, R. K, *et al.* Relation between brain displacement and local cerebral blood flow in patients with chronic subdural haematoma. *J Neurol Neurosurg Psychiatry* 2001;**71**:741–6.
18 Benyamin R, Trescot AM, Datta S, *et al.* Opioid complications and side effects. *Pain Physician* 2008;**11**:S105–20.
19 Sakabe T, Matsumoto M. Effects of anesthetic agents and other drugs on cerebral blood flow, metabolism, and intracranial pressure. In: Cottrell J, Young W, eds. *Cottrell and Young's Neuroanesthesia*. Philadelphia: : Elsevier 2010. 78–94.
20 Manninen P, Mehta J, Venkatraghavan L, *et al.* Comparison of propofol and volatile agents for maintenance of anesthesia during elective craniotomy procedures: systematic review and meta-analysisComparaison entre propofol et agents volatils pour le maintien de l'anesthésie pendant les interventions de. *Can J Anesth* 2014;**61**:347–56. doi:10.1007/s12630-014-0118-9
21 Schofield-Robinson OJ, Alderson P, Miller D, *et al.* Intravenous versus inhalational maintenance of anaesthesia for postoperative cognitive outcomes in elderly people undergoing non-cardiac surgery. *Cochrane Database Syst Rev* Published Online First: 2018. doi:10.1002/14651858.cd012317.pub2
22 Stubbs D, Jl B, Murray R, *et al.* Development and validation of an electronic post-operative morbidity score (EPOMS). *Anesth Analg* 2019;**129(4)**:935–42. doi:10.1213/ANE.0000000000003953
23 Brasel KJ, Lim HJ, Nirula R, *et al.* Length of Stay. *Arch Surg* 2007;**142**:461. doi:10.1001/archsurg.142.5.461
24 Office for National Statistics. 2011 Census - Population and Household Estimates for England and Wales. 2012;:1–36.http://www.ons.gov.uk/ons/dcp171778_270487.pdf (accessed 14 Nov 2019).
25 Association of Anaesthetists of Great Britan and Ireland. Recommendations for standards of monitoring during anaesthesia and recovery 2015. Published Online First: 2015. doi:10.1111/anae.13316/full
26 WHO Collaborating Centre for Drug Statistics Methodology. ATC/DDD Index 2019. 2019.https://www.whocc.no/atc_ddd_index/ (accessed 3 May 2019).
27 Patanwala AE, Duby J, Waters D, *et al.* Opioid conversions in acute care. *Ann Pharmacother* 2007;**41**:255–67. doi:10.1345/aph.1H421
28 Dickerman R, Reynolds A, Williamson J, *et al.* Guidelines for the management of severe traumatic brain injury, fourth edition. 2016. doi:10.1093/neuros/nyx308
29 Roughead EE, Pratt NL, Kemp-Casey A, *et al.* The validity of the Rx-Risk Comorbidity Index using medicines mapped to the Anatomical Therapeutic Chemical (ATC) Classification System. *BMJ Open* 2018;**8**:e021122. doi:10.1136/bmjopen-2017-

For peer review only - http://bmjopen.bmj.com/site/about/guidelines.xhtml

021122
30 R Core Team. R: A Language and Environment for Statistical Computing. 2013.
31 White IR, Royston P, Wood AM. Multiple imputation using chained equations: Issues and guidance for practice. *Stat Med* 2011;**30**:377–99. doi:10.1002/sim.4067
32 Buuren S van, Groothuis-Oudshoorn K. mice: Multivariate Imputation by Chained Equations in R. 2019. doi:10.18637/jss.v045.i03
33 Tierney N, Cook D, McBain M, *et al.* naniar: Data Structures, Summaries, and Visualisation for Missing Data. R package version 0.4.2. 2019.
34 van Buuren S. Chapter 7: Multilevel multiple imputation. In: *Flexible Imputation of Missing Data*. CRC Press 2018.
35 Lukasiewicz AM, Grant RA, Basques BA, *et al.* Patient factors associated with 30-day morbidity, mortality, and length of stay after surgery for subdural hematoma: a study of the American College of Surgeons National Surgical Quality Improvement Program. *J Neurosurg* 2016;**124**:760–6. doi:10.3171/2015.2.JNS142721
36 Coulter IC, Kolias AG, Marcus HJ, *et al.* Proposal for a prospective multi-centre audit of chronic subdural haematoma management in the United Kingdom and Ireland. *Br J Neurosurg* 2014;**28**:199–203. doi:10.3109/02688697.2013.835378
37 Kelsey T, Cavendish W. Personalised health and care 2020: Using data and Technology to Transform Outcomes for Patients and Citizens. A framework for action. 2014. doi:10.1177/0272989X06295361
38 Maas AIR, Menon DK, Adelson PD, *et al.* Traumatic brain injury: integrated approaches to improve prevention, clinical care, and research. *Lancet Neurol* 2017;**16**:987–1048. doi:10.1016/S1474-4422(17)30371-X
39 Association of Anaesthetists of Great Britan and Ireland. Recommendations for the Safe Transfer of Patients with Brain Injury. London: 2006.
40 Poulsen FR, Halle B, Pottegard A, *et al.* Subdural hematoma cases identified through a Danish patient register: diagnosis validity, clinical characteristics, and preadmission antithrombotic drug use. *Pharmacoepidemiol Drug Saf* 2016;**25**:1253–62.

AUTHORS CONTRIBUTIONS

DJS – Concept, study design, data manipulation, primary analysis, manuscript preparation

BD – Study design, manuscript preparation and editing

TB – Study design, manuscript preparation and editing

AJ – Data access, study design, manuscript preparation and editing

PJH – Interpretation of findings, manuscript preparation and editing

DKM – Interpretation of findings, manuscript preparation and editing

AE – Statistical and data science advice and supervision, manuscript preparation editing

RMB – Data access, study design, supervision, manuscript preparation and editing

For peer review only

Data Sharing Statement:

The data were collected as part of routine care and accessed as part of an NHS service evaluation. As a result, the authors are not in a position to make the data freely available. The authors will entertain scientific collaborations with interested parties on application subject to data use agreement by the trust.

Competing Interests Statement:

Funding

DJS is supported by a Wellcome Trust Clinician PhD Fellowship (overarching grant number: 204017/Z/16/Z)

PJH is supported by the NIHR (Research Professorship, Cambridge BRC, Global Health Research Group on Neurotrauma) and the Royal College of Surgeons of England;

DKM is supported by the NIHR (through an Emeritus Senior Investigator appointment and the Cambridge NIHR Biomedical Research Centre, and funding for Global Health Research Group on Neurotrauma).

TB is supported by the NIHR Global Health Research Group on Neurotrauma at the University of Cambridge. Internal Reference: RG89187

For peer review only

Figure Captions

Figure 1: Proportion of inpatients with specific neurological complications, extracted from an electronic health record, after surgery for chronic subdural haematoma, by postoperative day (*n = 530)*

Figure 2: Proportion of inpatients with organ specific complications (as defined by the electronic postoperative morbidity score), after surgery for chronic subdural haematoma, by postoperative day (*n = 530*).

Figure 3: Kaplan-Meier curves for postoperative time to discharge, dichotomised by referral source, in a retrospective cohort of operated chronic subdural haematoma (*n = 530)*. CUH = patients admitted directly to the neurosurgical centre at Cambridge University Hospitals NHS Foundation Trust, Non-CUH = patients referred from other hospitals in the

region. Shaded areas = 95% confidence intervals, dashed lines indicate median postoperative length of stay by group.

For peer review only

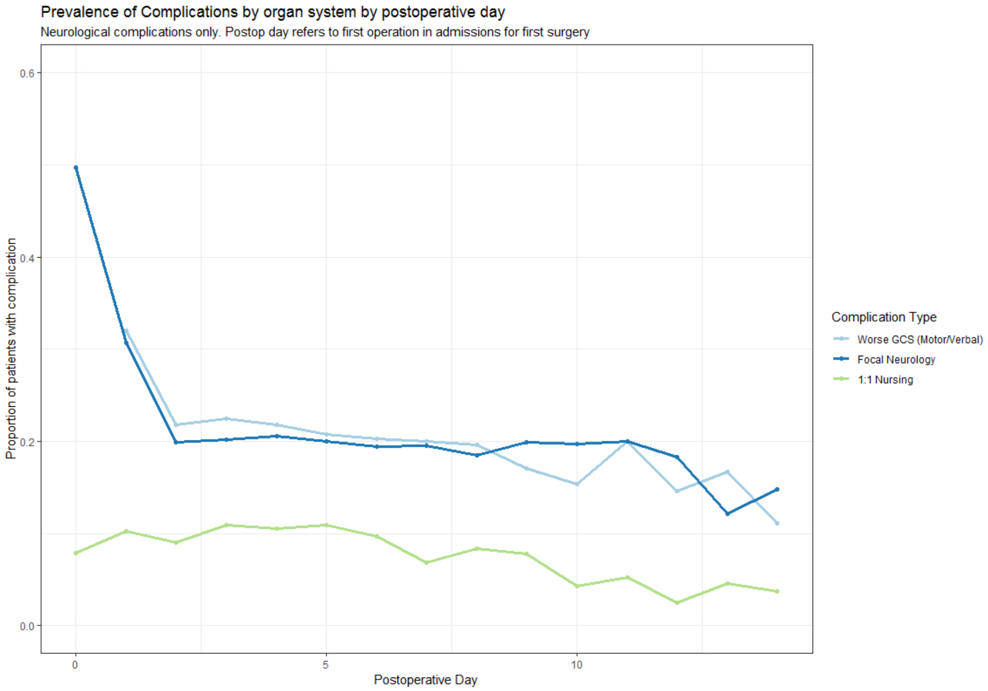

Figure 1: Proportion of inpatients with specific neurological complications, extracted from an electronic health record, after surgery for chronic subdural haematoma, by postoperative day (n = 530)

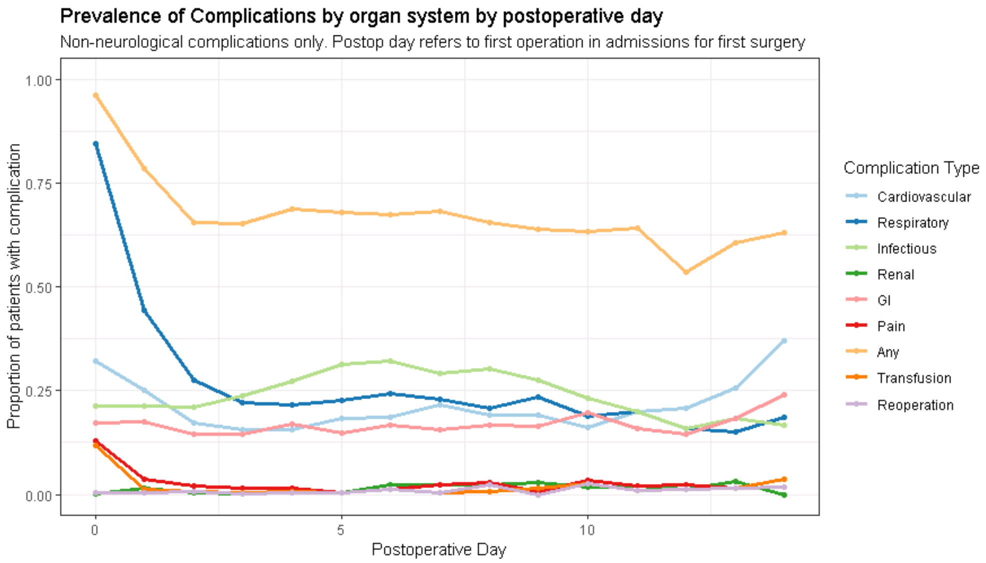

Figure 2: Proportion of inpatients with organ specific complications (as defined by the electronic postoperative morbidity score), after surgery for chronic subdural haematoma, by postoperative day (n = 530).

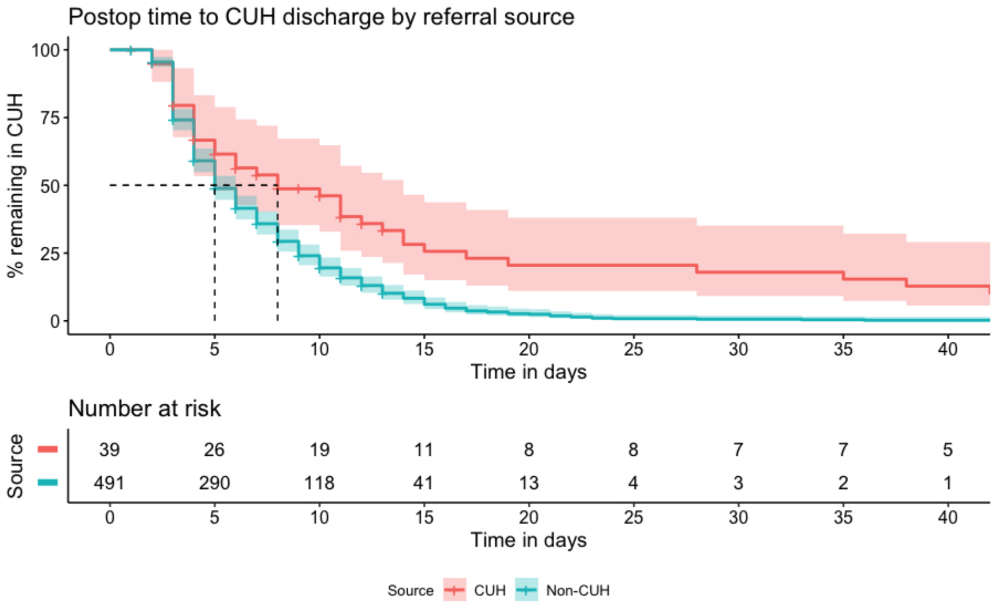

Figure 3: Kaplan-Meier curves for postoperative time to discharge, dichotomised by referral source, in a retrospective cohort of operated chronic subdural haematoma (n = 530). CUH = patients admitted directly to the neurosurgical centre at Cambridge University Hospitals NHS Foundation Trust, Non-CUH = patients referred from other hospitals in the region. Shaded areas = 95% confidence intervals, dashed lines indicate median postoperative length of stay by group.

319x198mm (72 x 72 DPI)

Supplementary Material: Impact of perioperative events extracted from an electronic health record on in-hospital length of stay after surgery for chronic subdural haematoma.

R-packages used in data manipulation and analysis

tidyverse[1]
mice[2]
naniar[3]
caret[4]
rlist[5]
plotROC[6]
ggsci[7]
survival[8]
survminer[9]
stargazer[10]

1 Wickham H. tidyverse: Easily Install and Load the 'Tidyverse'. R package version 1.2.1. https://cran.r-project.org/package=tidyverse%0A
2 Buuren S van, Groothuis-Oudshoorn K. mice: Multivariate Imputation by Chained Equations in R. 2019. doi:10.18637/jss.v045.i03
3 Tierney N, Cook D, McBain M, *et al.* naniar: Data Structures, Summaries, and Visualisation for Missing Data. R package version 0.4.2. 2019.https://cran.r-project.org/package=naniar
4 Kuhn M, et al. caret: Classification and Regression Training. R package version 6.0-84. https://cran.r-project.org/package=caret
5 Ren K. rlist: A Toolbox for Non-Tabular Data Manpiulation. R package version 0.4.6.1. 2016.
6 Sachs M. plotROC: A Tool for Plotting ROC Curves. Journal of Statistical Software. *J Stat Softw* 2017;**79**:1–19. doi:doi:10.18637/jss.v079.c02
7 Xiao N. ggsci: Scientific Journal and Sci-Fi Themed Color Palettes for 'ggplot2'. R package version 2.9. 2018.
8 T T. A package for Survival Analysis Version 2.38. 2015.
9 Kassambara Al. survminer: Drawing Survival Curves using 'ggplot2'. R package version 0.4.4.
10 Hlavac M. stargazer: Well-Formatted Regression and Summary Statistics Tables. R package version 5.2.1. 2018.

For peer review only

Definition of comorbidity

Comorbidity	WHO-ATC codes
**Anticoagulant/antiplatelet**	B01AC*, B01AA, B01AB, B01AD, B01AE, B01AF, B01AX
**Airways Disease**	R03*
**Heart Failure**	C07AB02, C07AB07, C07AG02, C07AB12, C03DA04 C03C*—C09*%
**History of CVS Disease**	C07AA, C07AB, C07AG01, C08C*, C08D*, C09DB, C09DX, C09BB C01DA, C01DX06, C08EX02

CVS = Cardiovascular, WHO-ATC = World Health Organisation Anatomical Therapeutic Classification code. Codes taken from the Rx-Risk score[65] * indicates that all drugs with codes below this level were counted as indicating this comorbidity. % for a diagnosis of heart failure based on drug codes C03C and C09 two drugs, one from each category must have been present to disentangle their use purely as anti-hypertensives. Anticoagulant use includes both antiplatelet and anticoagulants History of CVS disease was defined as those with angina or 'ischemic heart disease - hypertension' encoded in the Rx-Risk score.

Supplementary Table S1: *Patient comorbidities used in analysis and their calculation from a patient's prescribed medications, identified by World Health Organisation Anatomical Therapeutic Classification (WHO-ATC) code.*

Dictionary of WHO-ATC codes used in this analysis. Full details available at: https://www.whocc.no/atc_ddd_index/

A03FA01 = Metoclopramide
A04: Antiemetics and antinauseants

B01A: Antithrombotic agents

- B01AA = 'Vitamin K Antagonists'
- B01AB = Heparin Group
- **B01AC =** 'Platelet aggregation inhibitors excl. heparin'
- B01AD = Enzymes
- B01AE = Direct Thrombin Inhibitors
- B01AF = Direct Factor Xa inhibitors
- B01AX = Other antithrombotic agents

R03: Drugs for obstructive airway diseases

C07: Beta blocking agents

- *C07AB02 = Metoprolol*
- *C07AB07 = Bisoprolol*
- *C07AG02 = Carvedilol*
- *C07AB12 = Nebivolol*

C03C – C09:High-ceiling diuretics

For peer review only

C03DA04 = Eplerenone

C07AA = Beta blockers, non-selective
C07AB = Beta blockers, selective
C07AG01 = Labetalol
C08C = Calcium channel blockers with mainly vascular effects
C08D = Calcium channel blockers with direct cardiac effects
C09DB = Angiotensin II receptor blockers and calcium channel blockers
C09DX = Angiotension II receptor blockers, other combinations
C09BB = Ace inhibitors, calcium channel blockers
C01DA = Organic Nitrates
C01DX06 – Hexobendine
C08EX02 = perhexiline

J01 = Antibacterials for systemic use

N01BB01 = Bupivacaine
N02AA01 = Morphine
N02AB03 = Fentanyl

N05AD08 = Droperidol

R06AE03 = Cyclizine

For peer review only

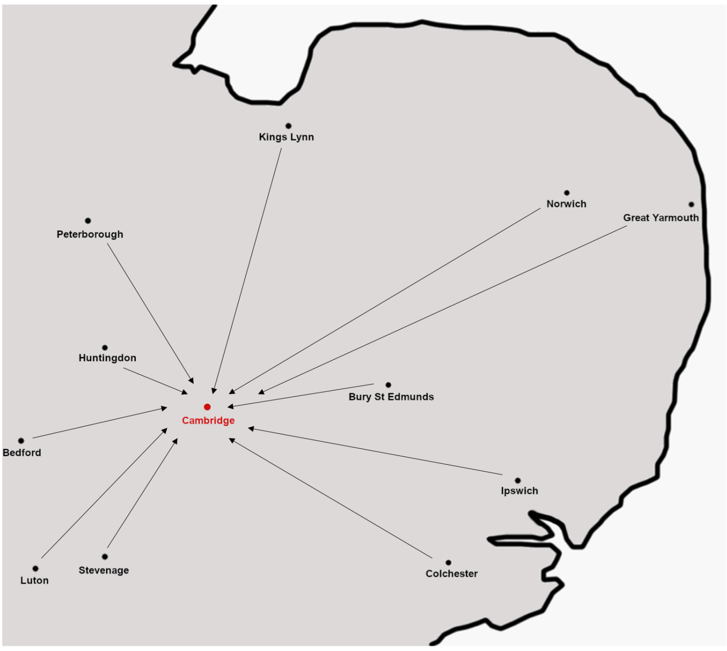

Referring hospitals are shown with black text, Cambridge neurosurgical centre shown in red. This only includes general referring hospitals although in reality, referrals are accepted from specialist hospitals such as Royal Papworth Hospital (Cardiac), and Broomfield Hospital (Plastics/Burns) that are not shown on this image.

Supplementary Figure S1: Referral region served by our neurosurgical centre.

For peer review only

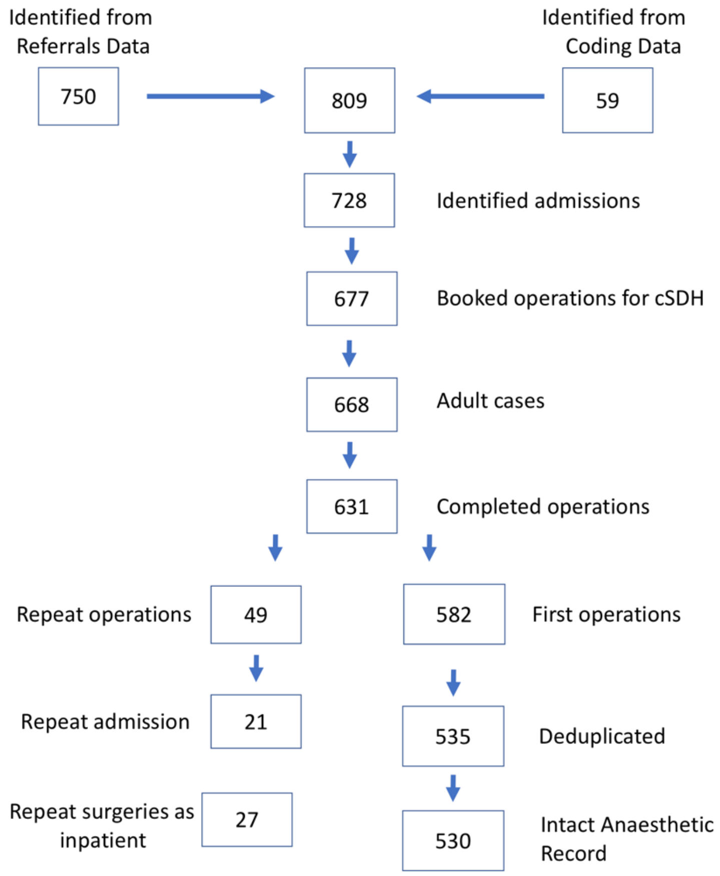

Supplementary Figure S2: **Identification of operated cases of chronic subdural haematoma at Cambridge University Hospitals NHS Foundation Trust (October 2014-January 2019).** CUH = Cambridge University Hospitals NHS Foundation Trust, cSDH = Chronic Subdural Haematoma. Cases from coding data are distinct individuals (from CUH) that were not found in the neurosurgical referrals database but were identified via diagnostic code. Discrepancy in number of admissions to number identified likely reflects cases that were eventually not transferred to CUH for surgery (for unknown reasons).

Missing Data patterns:

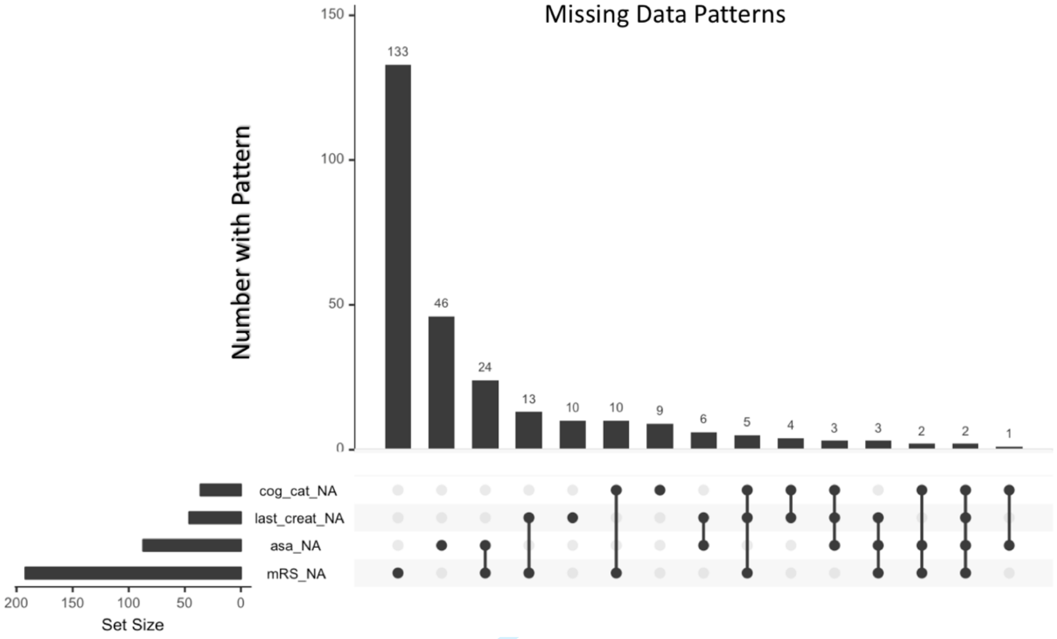

Supplementary Figure S3: Missing data patterns cog cat = presence of baseline cognitive impairment (Y/N), last creat = baseline creatinine measurement, asa = American Society of Anesthesiologists score, mRS = Modified Rankin Scale. Commonest pattern is that of missing mRS alone (n = 133). Vertical bars indicate number of individuals with each pattern (indicated by linked dots), horizontal bars indicate number of individuals with each missing variable.

Visualisation of Imputations

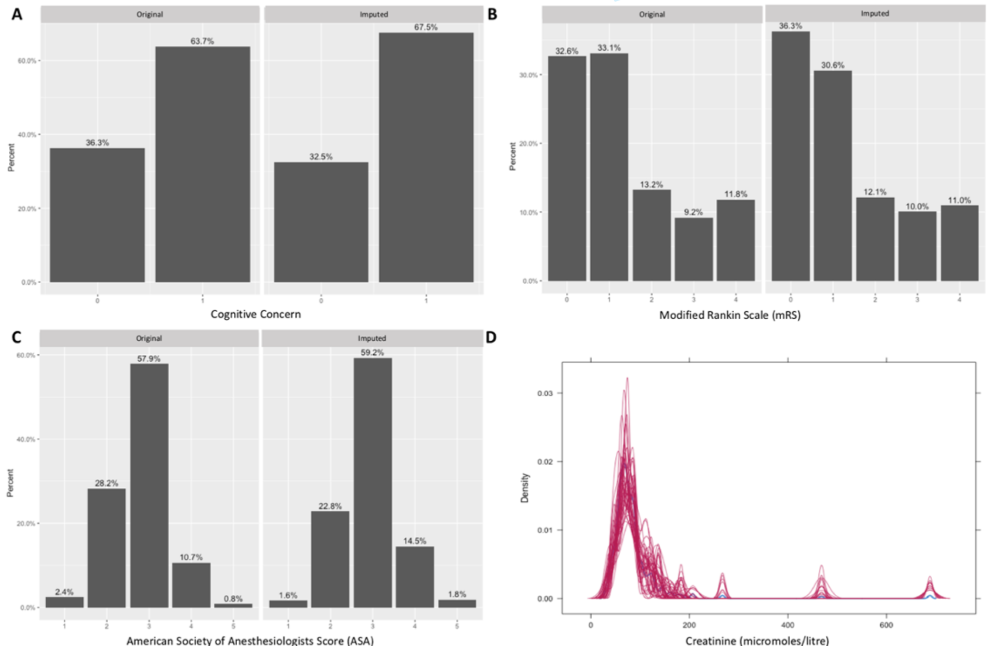

Supplementary Figure S4: *Visualisation of imputed values for A: Cognitive impairment documented on admission, B: Modified Rankin scale, C: American Society of Anesthesiologists (ASA) score, D: Creatinine.*

	HR	2.5 %	97.5 %	*p*
**Demographics**				
**Age** *Per year*	0.993	0.987	1.000	0.042*
**Sex** *Male v Female*	1.204	0.998	1.453	0.053*
**Non-CUH patient**	2.197	1.541	3.131	<0.001*
**ASA 2** *All v ASA 1*	0.488	0.298	0.798	0.004*
**ASA 3**	0.338	0.208	0.547	<0.001*
**ASA4**	0.250	0.141	0.444	<0.001
**ASA5**	0.200	0.058	0.686	0.011*
**mRS 1** *All v mRS 0*	0.813	0.627	1.053	0.117
**mRS2**	0.837	0.593	1.182	0.312
**mRS3**	0.781	0.517	1.179	0.239
**mRS4**	0.670	0.456	0.985	0.041*
**Admission status**				
**GCS 15** *on admission versus any-other*	1.109	1.056	1.164	<0.001*
**Admission ePOMS** *per 1 domain increase*	0.915	0.865	0.967	0.002*
**Cognitive impairment** *Yes/No*	0.558	0.466	0.667	<0.001
**Comorbidities**				
**Airways disease** *Yes/No*	0.747	0.584	0.956	0.020*
**CVS disease** *Yes/No*	0.834	0.702	0.991	0.039*
**Heart Failure** *Yes/No*	0.698	0.560	0.870	0.001*
**Creatinine** *per umol/l increase*	0.958	0.922	0.995	0.028*
**Anticoagulated on admission** *Yes/No*	0.417	0.349	0.498	<0.001*
**Day of Surgery variables**				
**Pre-op deterioration** *(Yes/No)*	1.066	0.890	1.276	0.488
**Length of wait** *Per hour*	0.963	0.917	1.013	0.142
**Time MAP <80mmHg** *per 1 minute*	0.996	0.973	1.020	0.720
**Time *ETCO2* not 3-5kPa** *per 1 minute*	0.982	0.916	1.053	0.616
**Fentanyl dose** *per microgram increase*	1.012	0.988	1.037	0.330
**Volatile maintenance** *Yes/No*	0.996	0.838	1.183	0.964
**Operative time** *per 1 minute*	0.971	0.951	0.990	0.004*
**Postoperative**				
**Complications***per 1 domain increase in ePOMS*	0.574	0.521	0.632	<0.001*

Complete Case Analyses: Univariable analysis

Supplementary Table S2: Results from univariable cox regression analysis performed in complete cases for time to postoperative discharge after surgery for chronic subdural haematoma*. Hazard ratios (HR) of greater than 1 indicate an association with more rapid postoperative discharge. Asterisks indicate statistical significance at a p<0.1, threshold chosen for progression to multivariable modelling. Postoperative complications included as a time dependent covariate (by day). 95% CI = 95% Confidence interval. CUH = ASA = American Society of Anesthesiologists score, CUH = Cambridge University Hospitals NHS Foundation Trust, CVS = cardiovascular, ePOMS = electronic postoperative morbidity score, $ETCO_2$ = End tidal carbon dioxide measurement, GCS = Glasgow coma scale, MAP*

For peer review only

= mean arterial pressure, mmHg = millimetres of mercury, mcg = micrograms, mRS = modified Rankin scale.

Complete Case Analysis: Multivariable analysis

	HR	95% CI	*p*-value
**Age** *(per year increase)*	0.99	0.98-1.01	0.301
**Sex** *(Male v Female)*	1.02	0.78-1.33	0.902
**GCS 15** *(v any-other value)*	1.03	0.93-1.15	0.581
**Referred patient**	2.40	0.69-8.30	0.167
**ASA** *(per 1 level increase)*	0.85	0.67-1.09	0.196
**Admission complications**	1.14	1.04-1.26	0.007*
**Airways disease** *(Yes/No)*	1.21	0.84-1.76	0.301
**CVS disease** *(Yes/No)*	1.13	0.84-1.52	0.424
**Anticoagulated on admission** *(Yes/No)*	0.52	0.40-0.69	<0.001*
**Heart failure** *(Yes/No)*	1.17	0.80-1.70	0.418
**mRS** *(per 1 level increase)*	1.03	0.93-1.13	0.566
**Baseline Creatinine** *(per 20umol/l increase)*	0.96	0.91-1.02	0.187
**Cognitive Concern** *(Yes/No)*	0.63	0.48-0.85	0.002*
**Operative time** *(per 10 minute increase)*	0.98	0.94-1.02	0.271
**Postoperative complications** *(per 1 domain increase in ePOMS)*	0.63	0.55-0.73	<0.001*

Supplementary Table S3: Results from multivariable cox regression model performed in complete cases (n = 260) for time to postoperative discharge after surgery for chronic subdural haematoma. *Hazard ratios (HR) of greater than 1 indicate association with a more rapid time to discharge. Asterisks indicate statistical significance at the 5% level. Postoperative complications included as a time dependent covariate (by day). 95% CI = 95% Confidence interval. CUH = ASA = American Society of Anesthesiologists score, CUH = Cambridge University Hospitals NHS Foundation Trust, CVS = cardiovascular, ePOMS = electronic postoperative morbidity score, GCS = Glasgow coma scale, mRS = modified Rankin scale.*

For peer review only

Sensitivity Analyses

	HR	95% CI	*p*-value
**Age** *(per year increase)*	0.99	0.99-1.00	0.169
**Sex** *(Male v Female)*	1.10	0.90-1.33	0.354
**Motor Score 6** *(v any-other value)*	0.97	0.83-1.12	0.674
**Referred patient**	1.53	1.01-2.29	0.042*
**ASA** *(per 1 level increase)*	0.88	0.75-1.04	0.135
**Admission complications**	1.05	0.99-1.12	0.102
**Airways disease** *(Yes/No)*	1.03	0.79-1.33	0.845
**CVS disease** *(Yes/No)*	0.96	0.78-1.19	0.715
**Anticoagulated on admission** *(Yes/No)*	0.45	0.37-0.54	<0.001*
**Heart failure** *(Yes/No)*	1.01	0.77-1.33	0.957
**mRS** *(per 1 level increase)*	0.99	0.91-1.08	0.835
**Baseline Creatinine** *(per 20umol/l increase)*	0.98	0.95-1.02	0.330
**Cognitive Concern** *(Yes/No)*	0.69	0.57-0.84	<0.001*
**Operative time** *(per 10 minute increase)*	0.99	0.97-1.01	0.374
**Postoperative complications** *(per 1 domain increase in ePOMS)*	0.61	0.55-0.68	<0.001*

Supplementary Table S4: Sensitivity analysis with total GCS replaced with an indicator variable for GCS motor score (6 or not) – highlighted in red.

For peer review only

	HR	95% CI	*p*-value
**Age** *(per year increase)*	0.99	0.99-1.00	0.046*
**Sex** *(Male v Female)*	1.15	0.94-1.39	0.168
**GCS 15** *(v any-other value)*	1.02	0.97-1.08	0.449
**Referred patient**	1.65	1.11-2.46	0.013*
**ASA** *(per 1 level increase)*	0.83	0.71-0.98	0.025*
**Admission complications**	1.01	0.95-1.07	0.841
**Airways disease** *(Yes/No)*	0.99	0.76-1.28	0.911
**CVS disease** *(Yes/No)*	0.96	0.78-1.19	0.732
**Anticoagulated on admission** *(Yes/No)*	0.45	0.37-0.54	<0.001*
**Heart failure** *(Yes/No)*	0.95	0.72-1.25	0.713
**mRS** *(per 1 level increase)*	0.97	0.89-1.07	0.561
**Baseline Creatinine** *(per 20umol/l increase)*	0.96	0.92-0.98	0.043*
**Cognitive Concern** *(Yes/No)*	0.65	0.53-0.79	<0.001*
**Operative time** *(per 10 minute increase)*	0.98	0.95-1.00	0.061

Supplementary Table S5: Sensitivity analysis, multivariable model with postoperative complication term excluded.

For peer review only

STROBE Statement—checklist of items that should be included in reports of observational studies

For: Utility of electronic health records in exploring associations between complex perioperative events morbidity and postoperative length of stay in surgically managed chronic subdural haematoma.
Stubbs et al.
(All page numbers refer to those in uploaded main word manuscript file)

	Item No.	Recommendation	Page No.	Relevant text from manuscript
**Title and abstract**	1	(*a*) Indicate the study's design with a commonly used term in the title or the abstract	2	
		(*b*) Provide in the abstract an informative and balanced summary of what was done and what was found	2	
**Introduction**				
Background/rationale	2	Explain the scientific background and rationale for the investigation being reported	3-4	
Objectives	3	State specific objectives, including any prespecified hypotheses	5	
**Methods**				
Study design	4	Present key elements of study design early in the paper	5	
Setting	5	Describe the setting, locations, and relevant dates, including periods of recruitment, exposure, follow-up, and data collection	5	
Participants	6	(*a*) *Cohort study*—Give the eligibility criteria, and the sources and methods of selection of participants. Describe methods of follow-up<br>*Case-control study*—Give the eligibility criteria, and the sources and methods of case ascertainment and control selection. Give the rationale for the choice of cases and controls<br>*Cross-sectional study*—Give the eligibility criteria, and the sources and methods of selection of participants	5	
		(*b*) *Cohort study*—For matched studies, give matching criteria and number of exposed and unexposed<br>*Case-control study*—For matched studies, give matching criteria and the number of controls per case		
Variables	7	Clearly define all outcomes, exposures, predictors, potential confounders, and effect modifiers.	6-8	

For peer review only - http://bmjopen.bmj.com/site/about/guidelines.xhtml

		Give diagnostic criteria, if applicable	
Data sources/ measurement	8*	For each variable of interest, give sources of data and details of methods of assessment (measurement). Describe comparability of assessment methods if there is more than one group	6-8
Bias	9	Describe any efforts to address potential sources of bias	Discussion
Study size	10	Explain how the study size was arrived at	5

Continued on next page

For peer review only

Quantitative variables	11	Explain how quantitative variables were handled in the analyses. If applicable, describe which groupings were chosen and why	8-9
Statistical methods	12	(*a*) Describe all statistical methods, including those used to control for confounding	8,
		(*b*) Describe any methods used to examine subgroups and interactions	
		(*c*) Explain how missing data were addressed	9 + Supplementary
		(*d*) *Cohort study*—If applicable, explain how loss to follow-up was addressed<br>*Case-control study*—If applicable, explain how matching of cases and controls was addressed<br>*Cross-sectional study*—If applicable, describe analytical methods taking account of sampling strategy	N/A
		(*e*) Describe any sensitivity analyses	11-12
**Results**			
Participants	13*	(a) Report numbers of individuals at each stage of study—eg numbers potentially eligible, examined for eligibility, confirmed eligible, included in the study, completing follow-up, and analysed	9 + Supplementary
		(b) Give reasons for non-participation at each stage	Supplementary
		(c) Consider use of a flow diagram	Supplementary
Descriptive data	14*	(a) Give characteristics of study participants (eg demographic, clinical, social) and information on exposures and potential confounders	10
		(b) Indicate number of participants with missing data for each variable of interest	9 + Supplementary
		(c) *Cohort study*—Summarise follow-up time (eg, average and total amount)	
Outcome data	15*	*Cohort study*—Report numbers of outcome events or summary measures over time	*9 + Fig 1*
		Case-control study—Report numbers in each exposure category, or summary measures of exposure	
		Cross-sectional study—Report numbers of outcome events or summary measures	
Main results	16	(*a*) Give unadjusted estimates and, if applicable, confounder-adjusted estimates and their precision (eg, 95% confidence interval). Make clear which confounders were adjusted for and why they were included	11-14
		(*b*) Report category boundaries when continuous variables were categorized	N/A
		(*c*) If relevant, consider translating estimates of relative risk into absolute risk for a meaningful time period	

For peer review only - http://bmjopen.bmj.com/site/about/guidelines.xhtml

Continued on next page

For peer review only

Other analyses	17	Report other analyses done—eg analyses of subgroups and interactions, and sensitivity analyses	11-12
**Discussion**			
Key results	18	Summarise key results with reference to study objectives	14
Limitations	19	Discuss limitations of the study, taking into account sources of potential bias or imprecision. Discuss both direction and magnitude of any potential bias	14-16
Interpretation	20	Give a cautious overall interpretation of results considering objectives, limitations, multiplicity of analyses, results from similar studies, and other relevant evidence	17
Generalisability	21	Discuss the generalisability (external validity) of the study results	17
**Other information**			
Funding	22	Give the source of funding and the role of the funders for the present study and, if applicable, for the original study on which the present article is based	18

*Give information separately for cases and controls in case-control studies and, if applicable, for exposed and unexposed groups in cohort and cross-sectional studies.

Note: An Explanation and Elaboration article discusses each checklist item and gives methodological background and published examples of transparent reporting. The STROBE checklist is best used in conjunction with this article (freely available on the Web sites of PLoS Medicine at http://www.plosmedicine.org/, Annals of Internal Medicine at http://www.annals.org/, and Epidemiology at http://www.epidem.com/). Information on the STROBE Initiative is available at www.strobe-statement.org.

For peer review only

BMJ Open

Identification of factors associated with morbidity and post-operative length of stay in surgically managed chronic subdural haematoma using electronic health records: a retrospective cohort study

Journal:	*BMJ Open*
Manuscript ID	bmjopen-2020-037385.R1
Article Type:	Original research
Date Submitted by the Author:	24-Mar-2020
Complete List of Authors:	Stubbs, Daniel; Cambridge University, University Division of Anaesthesia; Cambridge University, Healthcare Design Group<br>Davies, Benjamin; University of Cambridge, Department of Clinical Neurosurgery<br>Bashford, Tom; University of Cambridge , NIHR Global Health Research Group for Neurotrauma<br>Joannides, Alexis; Addenbrooke's Hospital, Department of Clinical Neurosciences; University of Cambridge, NIHR Global Health Research Group on Neurotrauma<br>Hutchinson, Peter; University of Cambridge, Academic Neurosurgery<br>Menon, David; NIHR Global Health Research Group for Neurotrauma, University of Cambridge<br>Ercole, Ari; Cambridge University Hospitals NHS Foundation Trust,<br>Burnstein, Rowan; Cambridge University Hospitals NHS Foundation Trust, Neurocritical Care Department and Department of Anaesthesia
**Primary Subject Heading**:	Anaesthesia
Secondary Subject Heading:	Surgery, Epidemiology, Neurology
Keywords:	ANAESTHETICS, EPIDEMIOLOGY, NEUROSURGERY, STATISTICS & RESEARCH METHODS, Quality in health care < HEALTH SERVICES ADMINISTRATION & MANAGEMENT

SCHOLARONE™
Manuscripts

I, the Submitting Author has the right to grant and does grant on behalf of all authors of the Work (as defined in the below author licence), an exclusive licence and/or a non-exclusive licence for contributions from authors who are: i) UK Crown employees; ii) where BMJ has agreed a CC-BY licence shall apply, and/or iii) in accordance with the terms applicable for US Federal Government officers or employees acting as part of their official duties; on a worldwide, perpetual, irrevocable, royalty-free basis to BMJ Publishing Group Ltd ("BMJ") its licensees and where the relevant Journal is co-owned by BMJ to the co-owners of the Journal, to publish the Work in this journal and any other BMJ products and to exploit all rights, as set out in our licence.

The Submitting Author accepts and understands that any supply made under these terms is made by BMJ to the Submitting Author unless you are acting as an employee on behalf of your employer or a postgraduate student of an affiliated institution which is paying any applicable article publishing charge ("APC") for Open Access articles. Where the Submitting Author wishes to make the Work available on an Open Access basis (and intends to pay the relevant APC), the terms of reuse of such Open Access shall be governed by a Creative Commons licence – details of these licences and which Creative Commons licence will apply to this Work are set out in our licence referred to above.

Other than as permitted in any relevant BMJ Author's Self Archiving Policies, I confirm this Work has not been accepted for publication elsewhere, is not being considered for publication elsewhere and does not duplicate material already published. I confirm all authors consent to publication of this Work and authorise the granting of this licence.

For peer review only

Identification of factors associated with morbidity and post-operative length of stay in surgically managed chronic subdural haematoma using electronic health records: a retrospective cohort study

Stubbs DJ[1%], Davies B[2], Bashford T[3], Joannides AJ[4,8], Hutchinson PJ[5,8], Menon DK[6], Ercole A[7], Burnstein RM[7]

1: Wellcome Trust Clinical Research Fellow, Specialist Registrar in Anaesthesia, 3: Clinical Research Fellow NIHR Global Neurotrauma group, Specialist Registrar in Anaesthesia, 6: Professor, 7: Consultant in neuroanaesthesia and neurointensive care, University Division of Anaesthesia, Department of Medicine, Addenbrooke's Hospital, Hills Road, Cambridge, CB2 0QQ

2: Research fellow, Specialist registrar, 4: Senior Research Associate, Consultant, 5: Professor, Division of Academic Neurosurgery, Department of Clinical Neurosciences, University of Cambridge, Addenbrooke's Hospital, Hills Road, Cambridge, CB2 0QQ

8: NIHR Brain Injury MedTech Co-operative, Department of Clinical Neurosciences, University of Cambridge, Cambridge

%: Corresponding author. Address for correspondence: djs225@cam.ac.uk

Word Count (Excluding Abstract): 3949

For peer review only

ABSTRACT

Introduction

Chronic subdural haematoma (cSDH) tends to occur in older patients, often with significant comorbidity. The incidence and effect of medical complications as well as the impact of intraoperative management strategies are now attracting increasing interest.

Objectives:

We used electronic health record (EHR) data to study the profile of in-hospital morbidity and examine associations between various intraoperative events and postoperative stay.

Design, setting, and participants:

Single-centre, retrospective cohort of 530 cases of cSDH (2014-2019) surgically evacuated under general anaesthesia at a neurosciences centre in Cambridge, UK.

Methods and outcome definition:

Complications were defined using a modified electronic postoperative morbidity score. Association between complications and intraoperative care (time with mean arterial pressure (MAP) <80mmHg, time outside of end-tidal carbon dioxide ($ETCO_2$) range of 3-5kPa, maintenance anaesthetic, operative time, and opioid dose) on postoperative stay was assessed using Cox-regression.

Results:

53 (10%) of patients suffered myocardial injury, 24 (4.5%) suffered acute renal injury. On postoperative day 3 (D3) 280 (58% of remaining) inpatients suffered at least one complication. D7 rate was comparable (57%). Operative time was the only intraoperative event associated with postoperative stay (Hazard Ratio for discharge: 0.97 [95% Confidence Interval 0.95-0.99]). Postoperative complications were associated with longer postoperative stay (0.91 [0.87-0.97] *per* complication). On multivariable analysis, complications (0.61 [0.55-0.68]), anticoagulation (0.45 [0.37-0.54]), and cognitive impairment (0.71 [0.58-0.87]) were associated with time to discharge.

Conclusions

There is a high postoperative morbidity burden in this cohort which was associated with postoperative stay. We found no evidence of an association between intraoperative events and postoperative stay.

For peer review only - http://bmjopen.bmj.com/site/about/guidelines.xhtml

STRENGTHS AND LIMITATIONS OF THIS STUDY:

- First use of electronic health data to chart the time course of in-hospital complications in a neurosurgical cohort
- Use of multiple imputation to maximise power with clear comparison to complete case analysis for comparison
- Methodological approaches are scalable to other surgical and in-patient cohorts
- Main results limited by being a single centre, retrospective study and thus lack external validity

KEYWORDS.

- Anaesthetics
- Epidemiology
- Neurosurgery
- Statistics and Research methods
- Quality in health care

For peer review only

For peer review only - http://bmjopen.bmj.com/site/about/guidelines.xhtml

INTRODUCTION

Chronic subdural haematoma (cSDH) is a common neurosurgical pathology of encapsulated blood products beneath the dural membrane, driven by trauma and inflammation [1]. Although cSDH can be managed conservatively, surgical evacuation is favoured in the presence of neurological deficit. cSDH is predominantly a "disease of the elderly". It is also associated with frailty and co-morbidity [2–5]. Increased mortality at one year amongst individuals with a cSDH has led some to consider it a 'sentinel health event' [6]. In these respects there is a clear analogy with patients suffering a fracture of the neck of femur (NOF), a group whose outcomes have recently been improved by concerted efforts into the measurement, integration, and improvement of holistic perioperative care [7,8]. However with cSDH, although a similarly effective surgical intervention exists, there has, as yet, been no demonstration of a systematic improvement in patient outcomes through analogous process changes.

To date, the impact of non-neurological morbidity in patients with cSDH has received little attention [9]. The reasons for this are likely multifactorial, but crucially there are significant challenges in adequately capturing patient morbidity using standard methodological approaches, which typically rely on cross-sectional assessment at set time points [10]. However, there is some evidence to suggest that non-neurological morbidity may be a significant problem. In a nationwide audit of UK cSDH management, 14% of 787 surgically treated patients suffered a complication, of which pneumonia was the most common [11]. The prevalence of such complications appears to increase with age [12,13]. The experience from parallel surgical fields would suggest this morbidity has implications for both patient outcomes, and the delivery of cost-effective healthcare [14]. This highlights a need for a more detailed investigation into the relationship between perioperative morbidity and outcome in cSDH. An understanding of the impact of all stages of the perioperative pathway on patient outcome is vital if potential clinical and service benefits from tailored process change, analogous to those in the NOF population, are to be realised [8].

Surgery for cSDH is commonly performed under general anaesthesia (GA). Heterogeneity in anaesthetic practice has the potential to introduce variation in postoperative outcome, yet

For peer review only

For peer review only - http://bmjopen.bmj.com/site/about/guidelines.xhtml

the impact of intraoperative events on outcome has received little attention in cSDH. Physiological changes, such as hypotension, can be a direct pharmacodynamic consequence of GA. Importantly, even brief periods below a mean arterial pressure (MAP) of 80mmHg are associated with end-organ damage [15], and it is reasonable to assume that the cSDH population may be particularly vulnerable. Furthermore, arterial carbon dioxide tension ($PaCO_2$) is no longer under homeostatic control during mechanical ventilation, which may have important implications for cerebral blood flow (CBF) and intracranial pressure (ICP) [16]. In cSDH, changes in CBF correlate with clinical symptoms [17]. GA choice may also have postoperative effects. Opioids have numerous side effects that occur with increasing frequency in the elderly [18]. The varied pharmacodynamic effects of anaesthetic agents have the potential to differentially impact on both ICP and CBF [19], with intravenous anaesthesia associated with lower rates of certain complications such as nausea and vomiting [20]. However, a recent systematic review (of low quality evidence) failed to demonstrate a difference in delirium rate or length of stay between patients receiving a volatile or intravenous anaesthetic [21].

The advent of integrated electronic health records (EHR) allows the capture of complex events such as intraoperative physiology, and to comprehensive measurement of in-patient morbidity across the entirety of an admission. New scoring systems are required to generate computable phenotypes from such complex, routinely collected data [22]. One such score, an electronic variant of the widely used postoperative morbidity score (POMS) [10], has equivalent discriminative performance in identifying discharge complexity or prolonged length of stay (LOS) in a heterogeneous population of elderly surgical patients [22]. LOS is often critiqued as an outcome measure in improvement efforts due to the impact of non-clinical events [23]. The complex relationship between clinical and non-clinical events in determining LOS is likely to vary between patients with differing disease processes. Regardless, its utility from the perspective of hospital bed occupancy is inarguable; with mounting health service pressures bed utilisation and patient throughput with minimal accrued morbidity is of crucial importance.

For peer review only - http://bmjopen.bmj.com/site/about/guidelines.xhtml

The aim of this study was to use routinely collected EHR data to examine, for the first time, the impact of intraoperative events and medical complications on postoperative length of neurosurgical centre stay in patients undergoing surgery for cSDH.

METHODS

Cohort Selection

This was a single centre, retrospective evaluation of surgically treated cSDH at Cambridge University Hospitals NHS Foundation Trust (CUH) between 26th October 2014 and 5th January 2019. CUH offers neurosurgical and trauma services to a population of approximately 5.8 million in the East of England (**Supplementary Figure S1)** [24]. 809 cases of cSDH were identified from the centre's neurosurgical referrals database and operation log, coded contemporaneously by neurosurgeons. After excluding missing records, duplicates, and incorrect procedures, 530 patients were included in this retrospective cohort (**Supplementary Figure S2**). The majority of patients (491, 92.5%) were transferred from other hospitals (*n = 14*). Anonymised data for each case was extracted from the CUH EHR (Epic Systems Corporation, Verona, WI) by the centre's clinical informatics team. The study was performed as part of a local service improvement programme, with local service evaluation approval obtained in line with UK procedure (ref: PRN 7705).

Patient and Public Involvement Statement:

Due to the retrospective and evaluative nature of the project no formal patient and public involvement work was undertaken.

Local surgical practice

Local practice is relatively standardised and comparable to previously published literature [25]. Procedures are predominantly performed under GA via two burr-holes with subsequent irrigation and usage of a subdural drain on free-drainage. The drain is removed after 48 hours with patients referred back to their local hospital at this point if required. Standard post-operative care is performed on a designated neurosurgical ward. Physiological observations are measured at a frequency dependent on the patients

For peer review only

calculated 'Early Warning Score' (EWS) in line with UK national guidance [26] and at least twice within a 24 hour period for all inpatients. Mini-craniotomy is reserved for recurrence or complex collections. Due to the use of standard OPCS procedure codes for all forms of subdural drainage, specific surgical approach was unable to be resolved.

Covariate definitions

Perioperative medical complications

A variant of the electronic postoperative morbidity score (ePOMS) [22] was utilised to determine the presence of morbidity across 9 distinct domains, following key organ systems (respiratory, cardiovascular, neurological, renal, haematological, gastro-intestinal, infectious, reoperation/wound, and pain) **(Table 1)**. Recognising that ePOMS had not previously been employed in a neurosurgical population, the score was modified to capture a greater number of dimensions of neurological morbidity. These included a deterioration in motor or verbal components of the Glasgow coma scale (GCS) (from admission), and the presence of asymmetric motor power. Therefore, the total score for an individual on any given day ranged between 0 and 11. Electronic observation, drug, and laboratory result charts were extracted from the EHR and the daily ePOMS generated for each patient. Physiological criteria (e.g. heart rate) were fulfilled if 2 or more instances occurred on a given day compared to a single event for investigation and drug-based criteria.

**Domain**	**Diagnostic Criteria**	**Notes**
**Respiratory**	Need for supplementary oxygen	
**Cardiovascular**	HR > 100<br><br>SBP < 100<br><br>Positive Troponin Test	
**Neurological**	Need for 1:1 nursing observation<br><br>Motor/verbal score worse than referral	Surrogate for hyperactive delirium

For peer review only - http://bmjopen.bmj.com/site/about/guidelines.xhtml

	Focal neurology	Documented mismatch between left/right sided arm/leg strength at any stage
**Renal**	Rise in creatinine to $\geqslant$ 1.5x baseline	Last recorded creatinine prior to surgery
**GI**	Administered anti-emetic	Defined by the following WHO ATC codes: a04**, a03fa01, r06ae03, n05ad08
**Pain**	Need for IV opioids or local anaesthetic infusion on a given day	Drugs identified by following WHO ATC codes: n02aa01, n02ab03, n01bb01
**Recurrence%**	Reoperation	EPOMS originally identifies severe wound infection by need for further surgery. In this context reoperation for the same procedure is being used to identify re-accumulation of cSDH
**Infection**	Temperature $\geqslant$ 38°C<br>Receiving Antibiotics on a given day	Antibiotics defined by following WHO ATC codes: j01**
**Haematological**	Transfused with blood product	Including red cells, platelets, FFP, cryoprecipitate.

Table 1: Definitions of the adapted electronic postoperative morbidity score (EPOMS) used for daily assessment of complications in a retrospective cohort of operated chronic subdural haematoma. HR = Heart rate, SBP = Systolic blood pressure, GCS = Glasgow Coma Scale, GI = Gastrointestinal, WHO ATC = World Health Organisation Anatomical therapeutic chemical classification, IV = intravenous, FFP = fresh frozen plasma. If multiple potential criteria are listed then an individual is positive in that domain if any of these are met. % In the original EPOMS score this would correspond to the wound category. * indicates additional criterion included in this variant of EPOMS from those previously published. ** indicates that all drugs below this level of ATC code were included. A dictionary of relevant ATC codes is available in the supplementary material.

Intra-operative covariates

Automatically recorded physiological measurements (blood pressure, heart rate) and inspired/expired gas measurements were extracted from the EHR at minute resolution throughout GA. Blood pressure was measured both non-invasively (typically at a frequency

For peer review only

For peer review only - http://bmjopen.bmj.com/site/about/guidelines.xhtml

of more than every 5 minutes [27]) and, for a subset of patients, invasively from an arterial catheter. All MAP values were combined and linearly interpolated. Total time (in minutes) spent below a threshold of 80mmHg [15] was calculated. In an effort to reduce artefact, values of 0 or >200mmHg were excluded. Maintenance anaesthetic was deemed inhalational if there were non-zero values of an inhalational anaesthetic agent. Text labels of manually recorded drug administrations were converted using a lookup table matching drugs to their corresponding World Health Organisation anatomical therapeutic classification (WHO-ATC) code [28]. All administrations of a drug with a code of N02A (opioid class) were extracted along with their dose. All opioid administrations were expressed as fentanyl equivalents using published conversions [29] as this is the most commonly used opioid in local neuroanaesthetic practice. Brain injury guidelines suggest that ventilation is adjusted to keep the level of carbon dioxide measured in arterial blood ($PaCO_2$) less than 6 and above 3.5kPa [30]. As end tidal values underestimate the true arterial gas tension by between 0.5-1kPa, time above an $ETCO_2$ of 5kPa and time below an $ETCO_2$ of 3kPa was calculated to give a total time outside of optimal CO_2 range.

Other Covariates

To control for residual confounding, key admission characteristics were also extracted from the EHR and referral database. These included: age, sex, American society of Anesthesiologists (ASA) score, modified Rankin scale (mRS), admission location (direct or from a referring hospital), baseline neurological status (GCS 15 yes/no, motor score 6 yes/no), admission creatinine, and key comorbidities. Preoperative deterioration (a worsening between ePOMS score on day of admission and day of surgery) and length of wait for surgery were also controlled for. Cognitive state on admission was defined using an admission screening questionnaire that ascertains the presence of temporary or permanent cognitive impairment. This was collapsed into a binary definition of ‘any’ cognitive impairment *versus* none.

As the majority of patients referred for surgery had no previous admission on record the baseline list of comorbidities was frequently incomplete. Comorbidities were inferred from admission medications using the ‘Rx-Risk’ score which uses WHO-ATC codes to determine

For peer review only - http://bmjopen.bmj.com/site/about/guidelines.xhtml

the presence of specific comorbidities [31]. This approach was chosen over discharge clinical coding to avoid potential reverse causation (e.g. in-hospital myocardial infarction (MI) being wrongly interpreted as a pre-existing history of myocardial infarction). Preadmission anticoagulant/antiplatelet use, airway disease, heart failure, or cardiovascular disease were determined using medication records reconciled in the first 24 hours of an individual's admission. Full codes used to determine comorbidity are shown in **Supplementary table S1.**

Statistical techniques

All analysis was performed in R (v 3.5.3) [32]. Effect of covariates on postoperative length of stay was performed using Cox regression. ePOMS scores were included as a time dependent covariate calculated per day, and all other variables were assumed to exert effect over the entire postoperative period. Univariable analysis was performed for all variables, those with a $p<0.1$ on initial regression were carried forward for multivariable modelling. At this stage statistical significance was taken as $p<0.05$. All analyses were conducted in both complete case and multiply imputed datasets.

Handling of missing data

Daily entries in the EHR for calculating ePOMS scores (such as blood results, observations, and administered drugs) were assumed to be accurate (e.g. if no troponin test was ordered a value was not imputed). Anaesthetic chart data (observations, measurements of gas tension) were also taken to be accurate due to automated data capture. Baseline patient variables (such as ASA score) were imputed using multiple imputation with chained equations (MICE) [33]. This was performed using the R packages 'mice'[34], and 'naniar'[35]. A full list of R packages used is available in the **supplementary material.** A multi-level imputation model, clustered by individual, was used to allow imputation of baseline data alongside longitudinal ePOMS scores [36]. Visualisation of missing data patterns are shown in **Supplementary Figure S3.** Four variables had missing data; baseline creatinine (missing in 46 – 8.7%), cognitive status (36 – 6.8%), ASA score (87 – 16.1%), and mRS (192 – 36.2%). All four were missing in only 2 cases. 40 imputed datasets were generated. Comparison of imputed and observed values can be seen graphically in **Supplementary Figure S4**. All results presented in the results reflect pooled analyses across

For peer review only - http://bmjopen.bmj.com/site/about/guidelines.xhtml

all imputed datasets, corresponding complete case analyses are in the **supplementary material (Tables S2 and S3).**

RESULTS

Cohort characteristics

These are summarised in **Table 2**, dichotomised by referral source. Direct admissions were younger (median age 74 *v* 77 *p=0.010*), and had fewer patients with a favourable motor score on admission (79.5% *v* 93.5% *p=0.001*). Time to surgery after admission to the tertiary centre was not significantly different but this did not account for any additional wait in referring hospitals (median = 10.6 [4.7-28.4] hours). Reoperation for recurrence was associated with significantly longer time to discharge (HR 0.38 *p<0.0001*).

Operative exposures

235 (44.2%) individuals received a volatile anaesthetic. 240 (45.2%) had a recorded entry of an intravenous anaesthetic. The majority of remaining cases likely received intravenous anaesthetic (due to the frequent presence of other anaesthetic drugs such as neuromuscular blockers on their drug chart) but could include a minority of cases performed under local anaesthetic.

Median duration of surgery was 96 minutes [IQR 78.0-103.5] with a maximum length of 318 minutes. Median time with a MAP < 80mmHg was 45 minutes [IQR: 25-69]. Median time outside of the optimal CO_2 range was 8 minutes [IQR: 4-15]. Median opioid dose in fentanyl equivalents was 100 micrograms (mcg) [IQR: 50-150]

Inpatient morbidity

13 individuals (2.4%) died during admission. 49 (9.2%) of patients were re-operated for their cSDH, 28 during their index admission. 53 (10%) of individuals exhibited some degree of postoperative troponin rise, with 24 (4.5%) developing acute kidney injury. 346 (65.2%) patients had a degree of preoperative deterioration between admission and surgery as determined by an increase in their ePOMS between these timepoints.

For peer review only - http://bmjopen.bmj.com/site/about/guidelines.xhtml

The prevalence of each complication subtype shown by postoperative day is demonstrated in **Figures 1 and 2**. There was an obvious restoration in focal neurology over the first two postoperative days **(Figure 1),** although no such trend was apparent in those needing 1:1 observation. Plots of incident complications in neurological, cardiovascular, respiratory, GI, pain, and infectious complications by day are shown in **Supplementary Figures S5 and S6.** Cases of new, worsening neurology after surgery were rare **(Supplementary Figure S5**), a pattern which differed from that of non-neurological complications (especially cardiovascular and respiratory) where new cases occurred throughout measured inpatient stay.

The apparently high prevalence of respiratory 'complications' on day of surgery and post-operative day 1 is likely spurious, reflecting the routine use of supplemental oxygen to counteract post-anaesthetic effects. Prevalence of infective complications appeared highest between days 3 and 10 (**Figure 2**). By day 3, 280 individuals (58% of 484 remaining inpatients) had at least one complication, a comparable rate to day 7 - 138 of 242 remaining inpatients (57%).

	**All (n=530)**	**Referred (n=491)**	**Local (n=39)**	***p***
**Variable**	**Median [IQR]**			
**Age** *years*	77 [69-84]	77 [70-84]	74 [62-79]	0.010*
**Creatinine** *μmol/l*	73 [61-89]	73 [61-89]	76 [64-100]	0.350
**Time to surgery (within centre)** *hours*	20.1 [9.5-40.3]	20.1 [9.8-42.7]	19.7 [5.2-35.9]	0.363
	***n* (%)**			
**Male**	376 (70.8)	349 (70.9)	27 (69.2)	0.855
**ASA ≥ 3**	271 (61.0)[%]	250 (61.2)	20 (57.1)	0.718
**Cognitively Impaired**	270 (54.5)[%]	254 (54.9)	16 (48.5)	0.485
**Admission GCS 15**	342 (64.4)	317 (64.5)	25 (64.1)	1
**Admission Motor Score 6**	490 (92.3)	459 (93.5)	31 (79.5)	0.001*
**mRS ≥ 2**	105 (31.0)[%]	104 (21.2)	1 (20.0)	1
**Anticoagulants/Antiplatelets**	232 (43.9)	211 (43.0)	21 (53.8)	0.241

For peer review only

**CVS disease**	238 (45.0)	225 (45.8)	13 (33.3)	0.136
**Heart failure**	101 (19.0)	90 (18.3)	11 (28.2)	0.139
**Airways disease**	75 (14.1)	66 (13.4)	9 (23.1)	0.099

Table 2: Baseline characteristics of retrospective cohort of operated chronic subdural haematoma (*n=530*). 'Referred' indicates cases referred from a regional hospital for surgery, 'Local' indicates a case admitted directly to the tertiary centre. ASA = American Society of Anesthesiologists score, GCS = Glasgow Coma Scale, mRS = Modified Rankin Score, CVS = Cardiovascular, Motor Score refers to score on the motor (movement) component of the Glasgow Coma Scale. ASA of 3 or more indicates presence of significant systemic comorbidity, modified Rankin scale of 2 or more indicates a level of disability that impedes normal activity. % indicates that value is calculated only on those with recorded values. *P* calculated for Mann Whitney U (continuous data) or Fisher's exact test (categorical). * indicates significant at 5% level.

For peer review only

Length of Stay

Overall median length of stay in the tertiary centre was 6.7 [IQR:4.2-10.6] days. For directly admitted patients this was 8.9 [IQR:5.2-16.3] days *versus* 6.5 [IQR:4.1-10.1] days for those referred from elsewhere. Post-operative length of stay was 4.9 [3.0-8.8] days in referred patients, compared to 7.9 [3.4-15.5] days in local patients. Postoperative time to discharge, dichotomised by referral source is shown in **Figure 3.**

Association of intra-operative events and complications on post-operative length of stay. From examined operative variables only operative time demonstrated a significant association (HR 0.97 [0.95-0.99]) on univariable analysis. Post-operative complications were associated with prolonged post-operative stay (HR 0.57 [0.52-0.63]) when included as a time dependent covariate. Baseline variables including age, sex, ASA score, mRS, medical state, and comorbidities were associated with longer time to post-operative discharge (**Table 3),** whilst a favourable GCS and being from a referred hospital was associated with a more rapid discharge from the tertiary centre.

18 of these variables were taken forward to multivariable modelling (**Table 4**). This was done to maximise control of confounding. Postoperative complications (HR 0.61 [0.55-0.68]), cognitive concerns (HR 0.71 [0.58-0.87]), and anticoagulation (HR 0.45 [0.37-0.54]) were all associated with longer time to discharge. Admission GCS (HR 1.03 [0.97-1.08]) and referral source (HR 1.46 [0.98-2.17]) were not significant in the final model. On complete

For peer review only - http://bmjopen.bmj.com/site/about/guidelines.xhtml

case analysis the pattern was similar except for an identified association with more rapid discharge with higher admission complication (ePOMS) scores (HR complete case 1.14 [1.04-1.26] *vs* HR MI 1.06 [0.99-1.12]) (**Supplementary Table S3**).

Based on these results, two sensitivity analyses were performed to attempt to understand the apparent lack of association between admission GCS and postoperative stay. Firstly, total GCS was replaced with an indicator for motor score (6 or not) (**Supplementary Table S4**) and secondly, a potential mediating effect of postoperative complications was examined by removing this covariate (**Supplementary Table S5)**. In the first analysis, motor score was not associated with post-operative time to discharge. In the second analysis no significant association with GCS was revealed in the subsequent model, although significant associations with admission ASA score, renal function, and age did become apparent (**Supplementary Table S5**).

For peer review only

	**HR**	**95% CI**	***p*-value**
**Demographics**			
**Age** *(per year increase)*	0.99	0.99 – 0.99	*0.042**
**Sex** *(male v female)*	1.20	0.99-1.45	*0.053**
**Referred patient**	2.20	1.54-3.13	*<0.001**
**ASA 2** *(All v ASA 1)*	0.50	0.31-0.82	*0.006**
**ASA 3**	0.34	0.21-0.55	*<0.001**
**ASA 4**	0.26	0.15-0.45	*<0.001**
**ASA 5**	0.22	0.07-0.68	*0.009**
**mRS 1** *(All v mRS 0)*	0.86	0.67-1.11	*0.249*
**mRS 2**	0.83	0.58-1.18	*0.299*
**mRS 3**	0.73	0.47-1.11	*0.142*
**mRS 4**	0.68	0.46-0.98	*0.040**
**Admission Status**			
**GCS 15 on admission** *(v any-other)*	1.11	1.06-1.16	*<0.001**
**Admission ePOMS** *(per 1 domain increase)*	0.91	0.87-0.97	*0.002**
**Cognitive impairment** *(yes/no)*	0.57	0.48-0.69	*<0.001**

For peer review only - http://bmjopen.bmj.com/site/about/guidelines.xhtml

**Comorbidities**			
**Airways disease**	0.75	0.58-0.96	*0.020**
**CVS disease**	0.83	0.70-0.99	*0.040**
**Heart Failure**	0.70	0.56-0.87	*0.001**
**Anticoagulated on admission**	0.42	0.35-0.50	*<0.001**
**Creatinine** (*per 20umol/l increase*)	0.96	0.92-0.99	*0.022**
**Day of Surgery**			
**Preop deterioration** *(Yes/No)*	1.07	0.89-1.28	*0.488*
**Length of wait** *(per hour)*	0.96	0.92-1.01	*0.142*
**Time MAP <80mmHg** (*per 10 minute increase*)	0.99	0.97-1.02	*0.720*
**Time ETCO$_2$ not 3-5kPa** *(per 10 minute increase)*	0.98	0.92-1.05	*0.616*
**Fentanyl dose** *(per 25 mcg increase)*	1.01	0.99-1.04	*0.330*
**Volatile maintenance**	0.99	0.84-1.18	*0.964*
**Operative time** *(per 10 minute increase)*	0.97	0.95-0.99	*0.004**
**Postoperative**			
**Complications** (*per 1 domain increase in ePOMS)*	0.57	0.52-0.63	*<0.001**

Table 3: Results from univariable cox regression of effects of specific variables on postoperative length of stay in a cohort of patients undergoing surgery for cSDH (*n =530)* pooled across *m=40* imputed datasets. Hazard ratios (HR) of more than 1 indicate a more rapid time to postoperative discharge. Asterisks indicate statistical significance at *p<0.1*, chosen threshold for inclusion in subsequent multivariable modelling. Postoperative complications included as a time dependent covariate (by day). 95% CI = 95% Confidence interval. ASA = American Society of Anesthesiologists score, CVS = cardiovascular, ePOMS = electronic postoperative morbidity score, ETCO$_2$ = End tidal carbon dioxide measurement, GCS = Glasgow coma scale, MAP = mean arterial pressure, mmHg = millimetres of mercury, mcg = micrograms, mRS = modified Rankin scale.

For peer review only

Multivariable analysis

	HR	95% CI	*p*-value
**Age** *(per year increase)*	0.99	0.99-1.00	0.114
**Sex** *(Male v Female)*	1.09	0.90-1.33	0.351
**GCS 15** *(v any-other value)*	1.03	0.97-1.08	0.377
**Referred patient**	1.46	0.98-2.17	0.063
**ASA** *(per 1 level increase)*	0.90	0.77-1.06	0.217
**Admission complications**	1.06	0.99-1.12	0.093
**Airways disease** *(Yes/No)*	1.03	0.79-1.34	0.819
**CVS disease** *(Yes/No)*	0.96	0.77-1.18	0.676
**Anticoagulated on admission** *(Yes/No)*	0.45	0.37-0.54	<0.001*
**Heart failure** *(Yes/No)*	1.02	0.77-1.33	0.908
**mRS** *(per 1 level increase)*	0.99	0.91-1.09	0.885
**Baseline Creatinine** *(per 20umol/l increase)*	0.98	0.94-1.01	0.242
**Cognitive Concern** *(Yes/No)*	0.71	0.58-0.87	<0.001*
**Operative time** *(per 10 minute increase)*	0.99	0.91-1.01	0.357
**Postoperative complications** *(per 1 domain increase in ePOMS)*	0.61	0.55-0.68	<0.001*

Table 4: Multivariable Cox regression model for postoperative time to discharge in a cohort of patients undergoing surgery for chronic subdural haematoma (*n* = 531), pooled across *m=40* imputed datasets. Hazard ratios (HR) of greater than 1 indicate association with a more rapid time to discharge. Asterisks indicate statistical significance at the 5% level. Postoperative complications included as a time dependent covariate (by day). 95% CI = 95% Confidence interval. ASA = American Society of Anesthesiologists score, CVS = cardiovascular, ePOMS = electronic postoperative morbidity score, $ETCO_2$ = End tidal carbon dioxide measurement, GCS = Glasgow coma scale, mRS = modified Rankin scale.

DISCUSSION

To our knowledge, this is the first study in cSDH to use routinely-collected EHR data to define postoperative morbidity and clinical interventions, such as anaesthetic events, whilst exploring their impact on LOS.

Principal findings

For peer review only

421 We highlight a significant degree of post-operative morbidity in surgically treated cSDH. Despite comparable cSDH recurrence rates of 9.2% (9% in a UK wide audit of practice [11]), in-patient morbidity captured by the ePOMS demonstrated a prevalence of medical complications of over 50% in the two weeks after surgery. 13% of patients demonstrated a rise in serum troponin or an episode of renal injury. Post-operative morbidity was significantly associated with longer tertiary centre LOS, raising the question of whether efforts to pre-empt or aggressively manage such morbidity could afford more efficient healthcare delivery and improved patient outcome. The total in-hospital burden of morbidity is likely higher than our estimate, as the single-centre nature of our study did not include complications occurring in a patient's referring hospital. Our study design also prevents us examining associations with total inpatient stay due to likely significant unmeasured events, including bed and transport availability. This data structure also precludes an assessment of mid-term mortality as records are not linked. Regardless, the presented findings are likely to be highly relevant to practice within tertiary neurosurgical centres.

436 **Baseline factors and confounding**

437 Cognitive function on admission, admission anticoagulation, and postoperative complications were all associated with longer time to discharge. Importantly, admission GCS was not associated with postoperative stay. One interpretation could be that any impact of pre-operative neurology on post-operative course may be mediated through the included post-operative complication term (e.g. low GCS is associated with length of stay through a predisposition to complications such as pneumonia). This explanation was not supported by a sensitivity analysis (**Supplementary Table S5**). A larger question is to consider which measure of neurological morbidity is best for prognostication in this patient cohort, both in terms of underlying pathophysiology and data coding. For instance, an individual with a hemiparesis arising from a non-dominant hemisphere lesion would likely be recorded as possessing a motor score of 6 and a GCS of 15 thus weakening any observed association. Unpicking these issues and the co-linearity inherent within these overlapping variables is difficult and should be explored in larger data sets.

For peer review only - http://bmjopen.bmj.com/site/about/guidelines.xhtml

We have attempted to control for many important variables identified in previous work in traumatic neurosurgical patients [37]. The significant change in the observed HR for complications between univariable and multivariable analysis (0.91 *v* 0.61) suggests we have controlled for significant negative confounders in the final model. The shift in HR for referral source on multivariable modelling suggests that at least some of its observed association with time to discharge reflects confounding or mediation by included variables, perhaps reflecting differences in patient cohorts (such as admission motor score or age – **Table 2**), or management between local and referred patients. These findings require further evaluation in future studies and distinct cohorts.

Comparison to other literature

Although not widely examined, studies that have examined medical complications in cSDH have suggested they may be a significant burden. A 2014 cross-sectional survey of UK neurosurgical centres demonstrated that 14% of 787 surgically treated cSDH suffered a complication. In this survey morbidity was defined as, '…any adverse event occurring during inpatient stay…' [38], with a predefined audit target of <10%. In our study, ePOMS defined morbidity peaked at 65.2%. This higher prevalence likely reflects the sensitivity of the scoring system, enabling the measurement of 'lower-level' morbidity not captured in previous studies. However, the consistent association of such ePOMS-defined morbidity with LOS indicates it is likely to be of clinical relevance. As EHRs and standardised data collection become more widespread, the utilisation of phenotyping techniques such as the ePOMS will become increasingly important but, in the short term, create a degree of difficulty in allowing direct comparisons to previously published literature.

Utility of EHRs in exploring peri-operative care

The evidence from our study is that interrogation of the EHR data may be a valuable addition to the improvement process, allowing the extraction and analysis of complex events and the use of computable phenotypes. This approach enables the evaluation of previously unmeasured clinical interactions on patient outcomes at different stages of the patient-journey. The importance of integrated care at a system level is widely recognised in

For peer review only

the care of traumatic brain injury. Here, interventions and further research can be prioritised based on an understanding of the drivers of outcome in each phase of care [39]. The nature of the cSDH referral pathway and the inherent complexity of the patient cohort means simple interventions to improve outcome are unlikely to be successful and a similar 'system' wide approach may be an appropriate response. Studies such as ours which examine hitherto unexplored events, will be essential for defining both the scope of the problem and areas for improvement.

In this study we have evaluated the impact of potentially significant elements of intra-operative care for the first time in a cohort of cSDH patients. Significant associations with LOS were not found. We defined covariates based on time outside optimal ranges, deviation from which is associated with adverse outcome in other surgical specialities [15], or recommended as physiologically appropriate in more severe head injury [30,40]. Periods of time (10 minutes or more) spent at a MAP of <80mmHg are associated with end-organ complications in non-cardiac surgery [15]. In the same setting, a 'dosing effect' is suggested, with shorter periods of time under lower MAP values associated with complications. 80mmHg was chosen for this project due to its recommendation in other settings of neurosurgical practice [40] but perhaps total time below this threshold, without considering a dosing effect may explain the lack of any observed association. Similarly, it may be that MAP targets should be individualised based on patient characteristics such as co-morbidities, and exploring such interactions represent a potential area of future research.

Limitations of EHR data

Utilising EHR-derived data has limitations, the majority of which are grounded in the availability of readily extractable data encapsulating key clinical characteristics such as radiological diagnoses or the specific events occurring during an operative procedure. The absence of information such as comorbidities required the use of novel phenotyping methods based on prescription medicines [31]. Although this methodology has been used in a large Australian database, the score has not previously, to our knowledge, been used in the surgical setting. Thus, novel elements of our methodology including drug-inferred

For peer review only

comorbidities and the ePOMS score require robust external validation in a distinct dataset despite apparent face validity. In larger cohorts, exploration of whether a weighted score (where different domains exert differing effects on LOS) could be explored. The need for such methods may vary by cohort and type of admission (e.g. emergency *v* elective). Hospital coding for SDH does not confidently delineate acute from chronic SDH [41], and operative codes do not encapsulate the specific technique used. In order to maintain anonymity of individual cases, we were not able to explore this by extracting free-text radiographic or operative details. Such analysis cannot be readily applied in a scaleable manner to allow automated calculation from aggregate data. Limitations on case definition in this study were mitigated through linkage with the centre's neurosurgical referral database, where cases are contemporaneously coded by a neurosurgical clinician, including a distinction between acute and chronic subdural haematoma. In addition, cases identified through coding data were refined by excluding those with associated major traumatic injuries to minimise the inclusion of cases of acute SDH. Despite these measures it is possible that some cases were incorrectly included.

Conclusion

This study provides the first comprehensive evaluation of inpatient care for surgically treated cSDH using routinely-collected data from an integrated EHR. Perioperative morbidity was common and associated with increased post-operative LOS. Pre-operative anticoagulation, admission GCS, cognitive dysfunction, and referral source were also associated with post-operative length of stay. This study identifies key areas to explore in quality improvement initiatives, whilst providing a methodological framework relevant to neurosurgery, as well as other surgical and inpatient specialities.

References

1 Edlmann E, Giorgi-Coll S, Whitfield PC, *et al.* Pathophysiology of chronic subdural haematoma: Inflammation, angiogenesis and implications for pharmacotherapy. *J Neuroinflammation* 2017;**14**:1–13. doi:10.1186/s12974-017-0881-y

2 Fogelholm R, Waltimo O. Epidemiology of chronic subdural haematoma. *Acta*

For peer review only

Neurochir (Wien) 1975;**32**:247–50.

3 Alexander E, Wagner E, Buchner D, *et al.* Do surgical brain lesions present as isolated dementia? A population- based study. *J Am Geriatr Soc* 1995;**43**:138–43.

4 Kudo H, Kuwamura K, Izawa I, *et al.* Chronic subdural hematoma in elderly people: present status on Awaji Island and epidemiological prospect. *Neurol Med Chir (Tokyo)* 1992;**32**:207–9.

5 Bartek J. J, Sjavik K, Stahl F, *et al.* Surgery for chronic subdural hematoma in nonagenarians: A Scandinavian population-based multicenter study. *Acta Neurol Scand* 2017;**136**:516–20.

6 Dumont TM, Rughani AI, Goeckes T, *et al.* Chronic subdural hematoma: A sentinel health event. *World Neurosurg* 2013;**80**:889–92.

7 Royal College of Physicians. *National Hip Fracture Database (NHFD) annual report 2017*. London: : Royal College of Physicians 2017. doi:ISBN978-1-86016-577-1

8 Gilchrist N, Dalzell K, Pearson S, *et al.* Enhanced hip fracture management: Use of statistical methods and dataset to evaluate a fractured neck of femur fast track pathway—pilot study. *N Z Med J* 2017;**130**:91–101.

9 Chari A, Hocking KC, Broughton E, *et al.* Core Outcomes and Common Data Elements in Chronic Subdural Hematoma: A Systematic Review of the Literature Focusing on Reported Outcomes. *J Neurotrauma* 2016;**33**:1212–9. doi:10.1089/neu.2015.3983

10 Grocott MPW, Browne JP, Van der Meulen J, *et al.* The Postoperative Morbidity Survey was validated and used to describe morbidity after major surgery. *J Clin Epidemiol* 2007;**60**:919–28. doi:10.1016/j.jclinepi.2006.12.003

11 Brennan PM, Kolias AG, Joannides AJ, *et al.* The management and outcome for patients with chronic subdural hematoma: a prospective, multicenter, observational cohort study in the United Kingdom. *J Neurosurg* 2016;**127**:732–9. doi:10.3171/2016.8.jns16134

12 Adhiyaman V, Chattopadhyay I, Irshad F, *et al.* Increasing incidence of chronic subdural haematoma in the elderly. *QJM-AN Int J Med* 2017;**110**:375–8. doi:10.1093/qjmed/hcw231

13 Munoz-Bendix C, Pannewitz R, Remmel D, *et al.* Outcome following surgical treatment of chronic subdural hematoma in the oldest-old population. *Neurosurg Rev* 2017;**40**:461–8. doi:10.1007/s10143-016-0803-y

14 National Confidential Enquiry into Perioperative Outcome & Death. An age old

For peer review only

problem. 2010. doi:10.1136/bmj.39356.408102.59

15 Wesselink EM, Kappen TH, Torn HM, *et al.* Intraoperative hypotension and the risk of postoperative adverse outcomes: a systematic review. *Br J Anaesth* 2018;**121**:706–21. doi:10.1016/j.bja.2018.04.036

16 Tameemm A, Krovvidi H. Cerebral physiology. *Contin Educ Anaesthesia, Crit Care Pain* 2013;**13**:113–8. doi:10.1093/bjaceaccp/mkt001

17 S. I, T. K, R. K, *et al.* Relation between brain displacement and local cerebral blood flow in patients with chronic subdural haematoma. *J Neurol Neurosurg Psychiatry* 2001;**71**:741–6.

18 Benyamin R, Trescot AM, Datta S, *et al.* Opioid complications and side effects. *Pain Physician* 2008;**11**:S105–20.

19 Sakabe T, Matsumoto M. Effects of anesthetic agents and other drugs on cerebral blood flow, metabolism, and intracranial pressure. In: Cottrell J, Young W, eds. *Cottrell and Young's Neuroanesthesia*. Philadelphia: : Elsevier 2010. 78–94.

20 Manninen P, Mehta J, Venkatraghavan L, *et al.* Comparison of propofol and volatile agents for maintenance of anesthesia during elective craniotomy procedures: systematic review and meta-analysisComparaison entre propofol et agents volatils pour le maintien de l'anesthésie pendant les interventions de. *Can J Anesth* 2014;**61**:347–56. doi:10.1007/s12630-014-0118-9

21 Schofield-Robinson OJ, Alderson P, Miller D, *et al.* Intravenous versus inhalational maintenance of anaesthesia for postoperative cognitive outcomes in elderly people undergoing non-cardiac surgery. *Cochrane Database Syst Rev* Published Online First: 2018. doi:10.1002/14651858.cd012317.pub2

22 Stubbs D, Jl B, Murray R, *et al.* Development and validation of an electronic post-operative morbidity score (EPOMS). *Anesth Analg* 2019;**129(4)**:935–42. doi:10.1213/ANE.0000000000003953

23 Brasel KJ, Lim HJ, Nirula R, *et al.* Length of Stay. *Arch Surg* 2007;**142**:461. doi:10.1001/archsurg.142.5.461

24 Office for National Statistics. 2011 Census - Population and Household Estimates for England and Wales. 2012;:1–36.http://www.ons.gov.uk/ons/dcp171778_270487.pdf (accessed 14 Nov 2019).

25 Santarius T, Kirkpatrick PJ, Ganesan D, *et al.* Use of drains versus no drains after burr-hole evacuation of chronic subdural haematoma: a randomised controlled trial. *Lancet* 2009;**374**:1067–73. doi:10.1016/S0140-6736(09)61115-6

For peer review only

26 National Institute for Health and Care Excellence. NICE Guideline CG50: Acutely ill adults in hospital: recognising and responding to deterioration. 2007.

27 Association of Anaesthetists of Great Britan and Ireland. Recommendations for standards of monitoring during anaesthesia and recovery 2015. Published Online First: 2015. doi:10.1111/anae.13316/full

28 WHO Collaborating Centre for Drug Statistics Methodology. ATC/DDD Index 2019. 2019.https://www.whocc.no/atc_ddd_index/ (accessed 3 May 2019).

29 Patanwala AE, Duby J, Waters D, *et al.* Opioid conversions in acute care. *Ann Pharmacother* 2007;**41**:255–67. doi:10.1345/aph.1H421

30 Dickerman R, Reynolds A, Williamson J, *et al.* Guidelines for the management of severe traumatic brain injury, fourth edition. 2016. doi:10.1093/neuros/nyx308

31 Roughead EE, Pratt NL, Kemp-Casey A, *et al.* The validity of the Rx-Risk Comorbidity Index using medicines mapped to the Anatomical Therapeutic Chemical (ATC) Classification System. *BMJ Open* 2018;**8**:e021122. doi:10.1136/bmjopen-2017-021122

32 R Core Team. R: A Language and Environment for Statistical Computing. 2013.

33 White IR, Royston P, Wood AM. Multiple imputation using chained equations: Issues and guidance for practice. *Stat Med* 2011;**30**:377–99. doi:10.1002/sim.4067

34 Buuren S van, Groothuis-Oudshoorn K. mice: Multivariate Imputation by Chained Equations in R. 2019. doi:10.18637/jss.v045.i03

35 Tierney N, Cook D, McBain M, *et al.* naniar: Data Structures, Summaries, and Visualisation for Missing Data. R package version 0.4.2. 2019.

36 van Buuren S. Chapter 7: Multilevel multiple imputation. In: *Flexible Imputation of Missing Data*. CRC Press 2018. available at: https://stefvanbuuren.name/fimd/

37 Lukasiewicz AM, Grant RA, Basques BA, *et al.* Patient factors associated with 30-day morbidity, mortality, and length of stay after surgery for subdural hematoma: a study of the American College of Surgeons National Surgical Quality Improvement Program. *J Neurosurg* 2016;**124**:760–6. doi:10.3171/2015.2.JNS142721

38 Coulter IC, Kolias AG, Marcus HJ, *et al.* Proposal for a prospective multi-centre audit of chronic subdural haematoma management in the United Kingdom and Ireland. *Br J Neurosurg* 2014;**28**:199–203. doi:10.3109/02688697.2013.835378

39 Maas AIR, Menon DK, Adelson PD, *et al.* Traumatic brain injury: integrated approaches to improve prevention, clinical care, and research. *Lancet Neurol* 2017;**16**:987–1048. doi:10.1016/S1474-4422(17)30371-X

For peer review only

40 Association of Anaesthetists of Great Britan and Ireland. Recommendations for the Safe Transfer of Patients with Brain Injury. London: 2006.

41 Poulsen FR, Halle B, Pottegard A, *et al.* Subdural hematoma cases identified through a Danish patient register: diagnosis validity, clinical characteristics, and preadmission antithrombotic drug use. *Pharmacoepidemiol Drug Saf* 2016;**25**:1253–62.

AUTHORS CONTRIBUTIONS

DJS – Concept, study design, data manipulation, primary analysis, manuscript preparation

BD – Study design, manuscript preparation and editing

TB – Study design, manuscript preparation and editing

AJ – Data access, study design, manuscript preparation and editing

PJH – Interpretation of findings, manuscript preparation and editing

DKM – Interpretation of findings, manuscript preparation and editing

AE – Statistical and data science advice and supervision, manuscript preparation editing

RMB – Data access, study design, supervision, manuscript preparation and editing

Data Sharing Statement:

The data were collected as part of routine care and accessed as part of an NHS service evaluation. As a result, the authors are not in a position to make the data freely available. The authors will entertain scientific collaborations with interested parties on application subject to data use agreement by the trust.

Conflicts of interest:

All authors assert no conflicts of interest.

Funding

DJS is supported by a Wellcome Trust Clinician PhD Fellowship (overarching grant number: 204017/Z/16/Z)

For peer review only - http://bmjopen.bmj.com/site/about/guidelines.xhtml

PJH is supported by the NIHR (Research Professorship, Cambridge BRC, Global Health Research Group on Neurotrauma) and the Royal College of Surgeons of England;

DKM is supported by the NIHR (through an Emeritus Senior Investigator appointment and the Cambridge NIHR Biomedical Research Centre, and funding for Global Health Research Group on Neurotrauma).

TB is supported by the NIHR Global Health Research Group on Neurotrauma at the University of Cambridge. Internal Reference: RG89187

AJJ is supported by the NIHR Brain Injury MedTech Co-operative based at the University of Cambridge.

The views are those of the authors and not necessarily those of the NHS, the NIHR, the Wellcome Trust, or the Department of Health.

Figure Captions

For peer review only

Figure 1: Proportion of inpatients with specific neurological complications, extracted from an electronic health record, after surgery for chronic subdural haematoma, by post-operative day (*n = 530)*

Figure 2: Proportion of inpatients with organ specific complications (as defined by the electronic postoperative morbidity score), after surgery for chronic subdural haematoma, by post-operative day (*n = 530*).

Figure 3: Kaplan-Meier curves for postoperative time to discharge, dichotomised by referral source, in a retrospective cohort of operated chronic subdural haematoma (*n = 530)*. CUH = patients admitted directly to the neurosurgical centre at Cambridge University Hospitals NHS Foundation Trust, Non-CUH = patients referred from other hospitals in the region. Shaded areas = 95% confidence intervals, dashed lines indicate median postoperative length of stay by group.

For peer review only

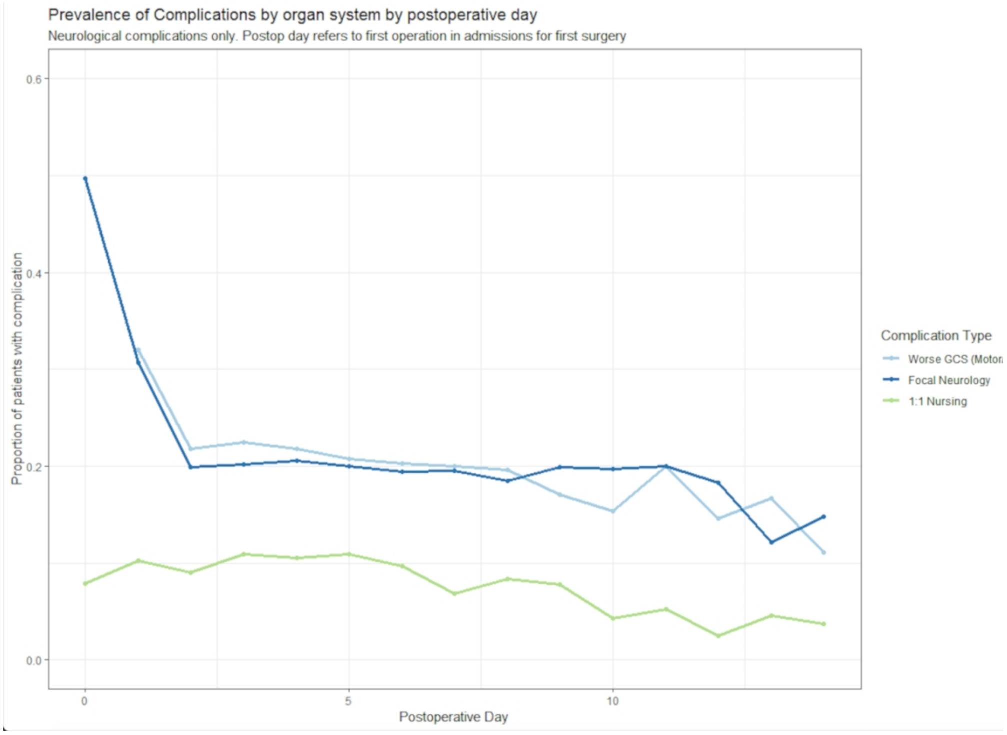

Figure 1: Proportion of inpatients with specific neurological complications, extracted from an electronic health record, after surgery for chronic subdural haematoma, by post-operative day (n = 530)

123x90mm (300 x 300 DPI)

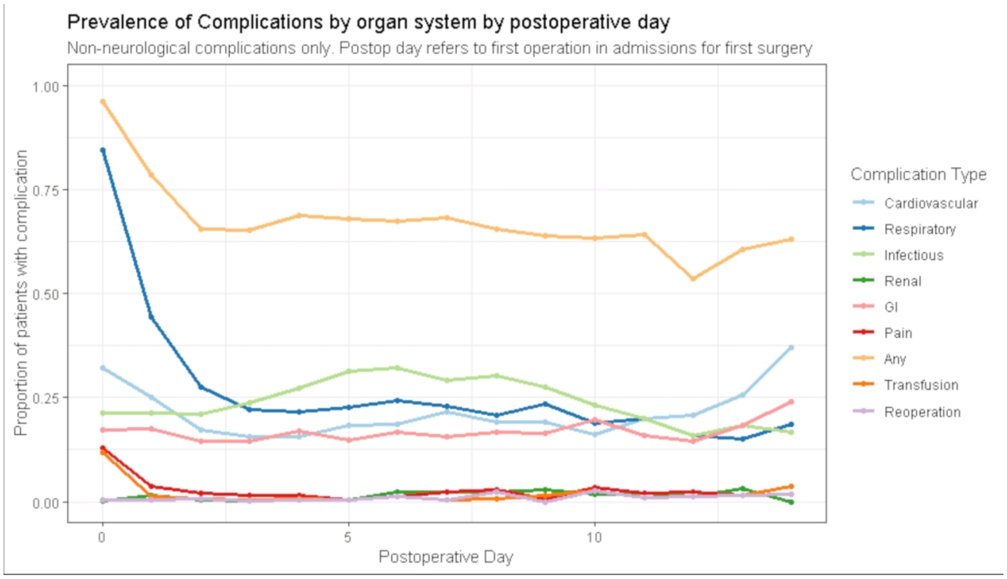

Figure 2: Proportion of inpatients with organ specific complications (as defined by the electronic postoperative morbidity score), after surgery for chronic subdural haematoma, by post-operative day (n = 530).

157x90mm (300 x 300 DPI)

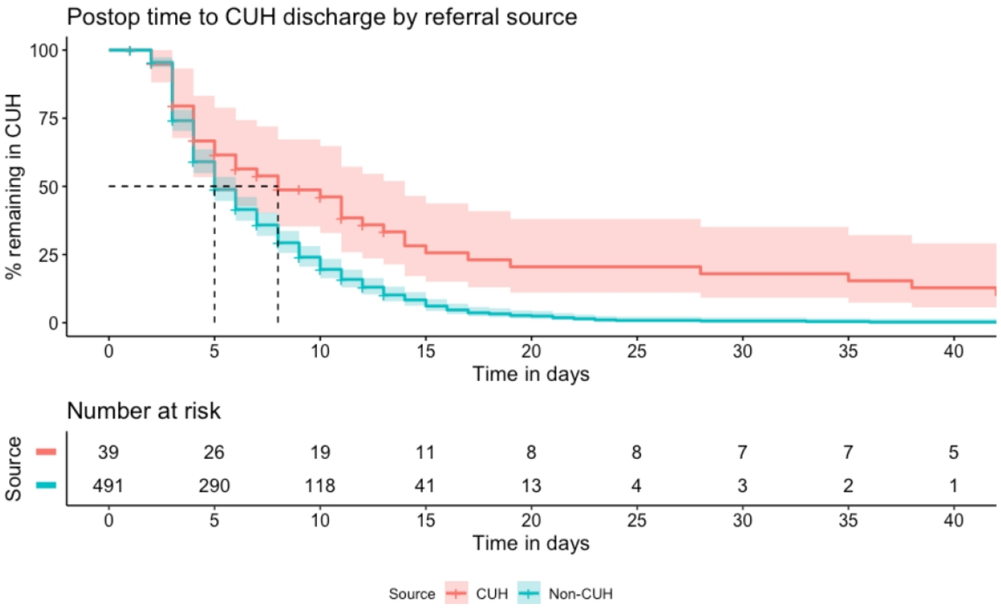

Figure 3: Kaplan-Meier curves for postoperative time to discharge, dichotomised by referral source, in a retrospective cohort of operated chronic subdural haematoma (n = 530). CUH = patients admitted directly to the neurosurgical centre at Cambridge University Hospitals NHS Foundation Trust, Non-CUH = patients referred from other hospitals in the region. Shaded areas = 95% confidence intervals, dashed lines indicate median postoperative length of stay by group.

152x91mm (300 x 300 DPI)

Supplementary Material: Impact of perioperative events extracted from an electronic health record on in-hospital length of stay after surgery for chronic subdural haematoma.

R-packages used in data manipulation and analysis

tidyverse[1]
mice[2]
naniar[3]
caret[4]
rlist[5]
plotROC[6]
ggsci[7]
survival[8]
survminer[9]
stargazer[10]

1 Wickham H. tidyverse: Easily Install and Load the 'Tidyverse'. R package version 1.2.1. https://cran.r-project.org/package=tidyverse%0A
2 Buuren S van, Groothuis-Oudshoorn K. mice: Multivariate Imputation by Chained Equations in R. 2019. doi:10.18637/jss.v045.i03
3 Tierney N, Cook D, McBain M, *et al.* naniar: Data Structures, Summaries, and Visualisation for Missing Data. R package version 0.4.2. 2019.https://cran.r-project.org/package=naniar
4 Kuhn M, et al. caret: Classification and Regression Training. R package version 6.0-84. https://cran.r-project.org/package=caret
5 Ren K. rlist: A Toolbox for Non-Tabular Data Manpiulation. R package version 0.4.6.1. 2016.
6 Sachs M. plotROC: A Tool for Plotting ROC Curves. Journal of Statistical Software. *J Stat Softw* 2017;**79**:1–19. doi:doi:10.18637/jss.v079.c02
7 Xiao N. ggsci: Scientific Journal and Sci-Fi Themed Color Palettes for 'ggplot2'. R package version 2.9. 2018.
8 T T. A package for Survival Analysis Version 2.38. 2015.
9 Kassambara Al. survminer: Drawing Survival Curves using 'ggplot2'. R package version 0.4.4.
10 Hlavac M. stargazer: Well-Formatted Regression and Summary Statistics Tables. R package version 5.2.1. 2018.

For peer review only

Definition of comorbidity

Comorbidity	WHO-ATC codes
**Anticoagulant/antiplatelet**	B01AC*, B01AA, B01AB, B01AD, B01AE, B01AF, B01AX
**Airways Disease**	R03*
**Heart Failure**	C07AB02, C07AB07, C07AG02, C07AB12, C03DA04 C03C*—C09*%
**History of CVS Disease**	C07AA, C07AB, C07AG01, C08C*, C08D*, C09DB, C09DX, C09BB C01DA, C01DX06, C08EX02

CVS = Cardiovascular, WHO-ATC = World Health Organisation Anatomical Therapeutic Classification code. Codes taken from the Rx-Risk score[65] * indicates that all drugs with codes below this level were counted as indicating this comorbidity. % for a diagnosis of heart failure based on drug codes C03C and C09 two drugs, one from each category must have been present to disentangle their use purely as anti-hypertensives. Anticoagulant use includes both antiplatelet and anticoagulants History of CVS disease was defined as those with angina or 'ischemic heart disease - hypertension' encoded in the Rx-Risk score.

Supplementary Table S1: *Patient comorbidities used in analysis and their calculation from a patient's prescribed medications, identified by World Health Organisation Anatomical Therapeutic Classification (WHO-ATC) code.*

Dictionary of WHO-ATC codes used in this analysis. Full details available at:
https://www.whocc.no/atc_ddd_index/

A03FA01 = Metoclopramide
A04: Antiemetics and antinauseants

B01A: Antithrombotic agents

- B01AA = 'Vitamin K Antagonists'
- B01AB = Heparin Group
- **B01AC =** 'Platelet aggregation inhibitors excl. heparin'
- B01AD = Enzymes
- B01AE = Direct Thrombin Inhibitors
- B01AF = Direct Factor Xa inhibitors
- B01AX = Other antithrombotic agents

R03: Drugs for obstructive airway diseases

C07: Beta blocking agents

- *C07AB02 = Metoprolol*
- *C07AB07 = Bisoprolol*
- *C07AG02 = Carvedilol*
- *C07AB12 = Nebivolol*

C03C – C09:High-ceiling diuretics

For peer review only

C03DA04 = Eplerenone

C07AA = Beta blockers, non-selective
C07AB = Beta blockers, selective
C07AG01 = Labetalol
C08C = Calcium channel blockers with mainly vascular effects
C08D = Calcium channel blockers with direct cardiac effects
C09DB = Angiotensin II receptor blockers and calcium channel blockers
C09DX = Angiotension II receptor blockers, other combinations
C09BB = Ace inhibitors, calcium channel blockers
C01DA = Organic Nitrates
C01DX06 – Hexobendine
C08EX02 = perhexiline

J01 = Antibacterials for systemic use

N01BB01 = Bupivacaine
N02AA01 = Morphine
N02AB03 = Fentanyl

N05AD08 = Droperidol

R06AE03 = Cyclizine

For peer review only

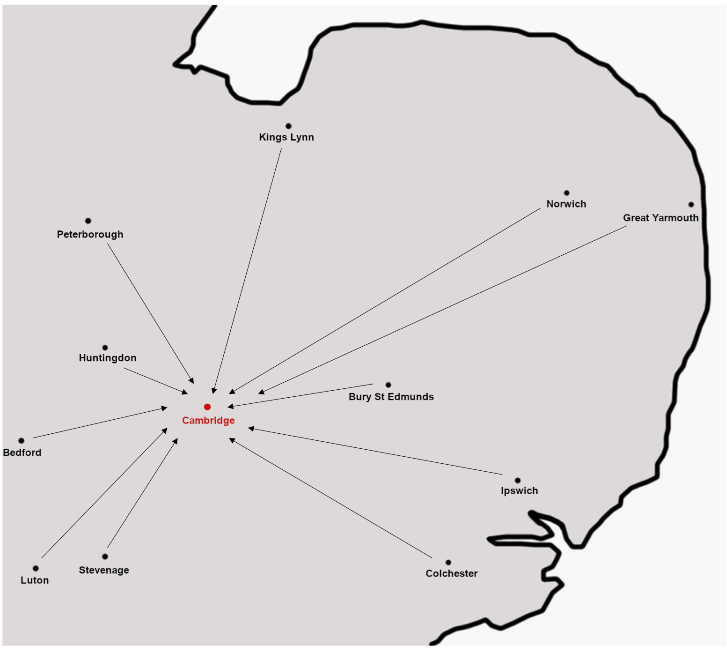

Referring hospitals are shown with black text, Cambridge neurosurgical centre shown in red. This only includes general referring hospitals although in reality, referrals are accepted from specialist hospitals such as Royal Papworth Hospital (Cardiac), and Broomfield Hospital (Plastics/Burns) that are not shown on this image.

Supplementary Figure S1: Referral region served by our neurosurgical centre.

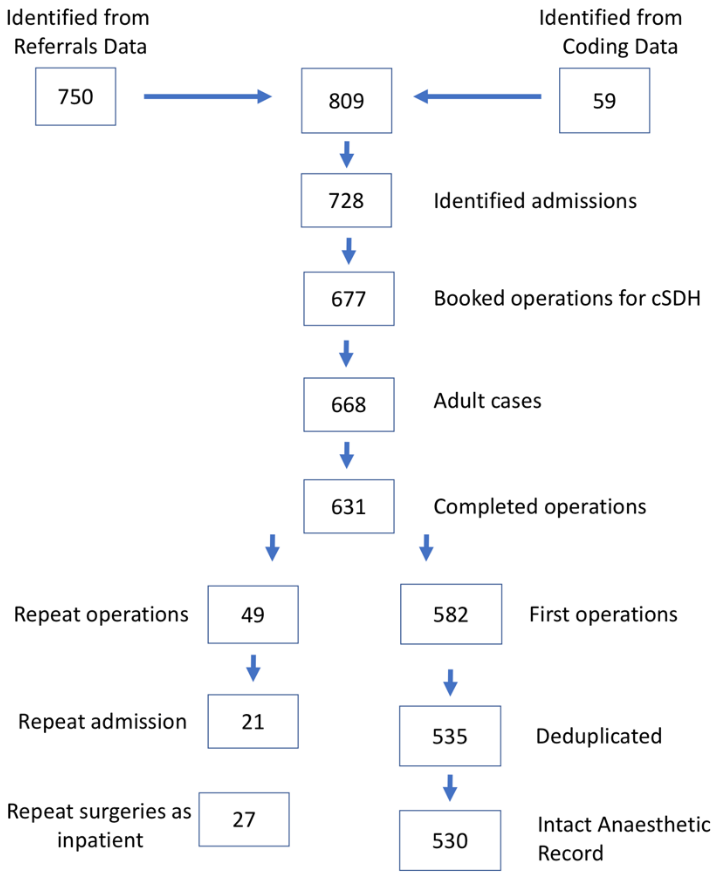

Supplementary Figure S2: **Identification of operated cases of chronic subdural haematoma at Cambridge University Hospitals NHS Foundation Trust (October 2014-January 2019).** CUH = Cambridge University Hospitals NHS Foundation Trust, cSDH = Chronic Subdural Haematoma. Cases from coding data are distinct individuals (from CUH) that were not found in the neurosurgical referrals database but were identified via diagnostic code. Discrepancy in number of admissions to number identified likely reflects cases that were eventually not transferred to CUH for surgery (for unknown reasons).

For peer review only - http://bmjopen.bmj.com/site/about/guidelines.xhtml

Missing Data patterns:

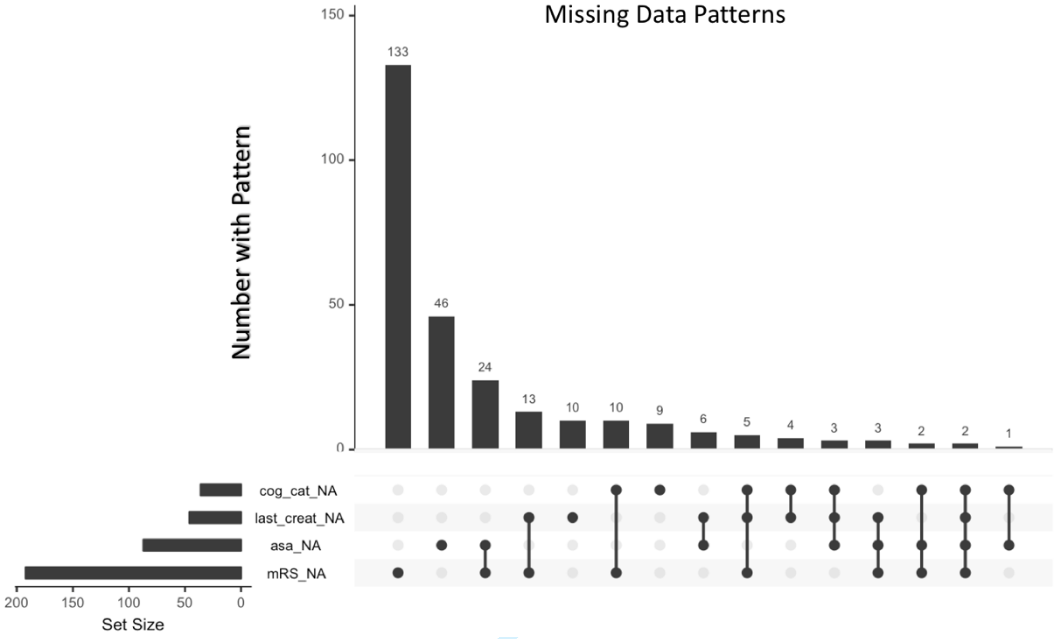

Supplementary Figure S3: Missing data patterns cog cat = presence of baseline cognitive impairment (Y/N), last creat = baseline creatinine measurement, asa = American Society of Anesthesiologists score, mRS = Modified Rankin Scale. Commonest pattern is that of missing mRS alone (n = 133). Vertical bars indicate number of individuals with each pattern (indicated by linked dots), horizontal bars indicate number of individuals with each missing variable.

Visualisation of Imputations

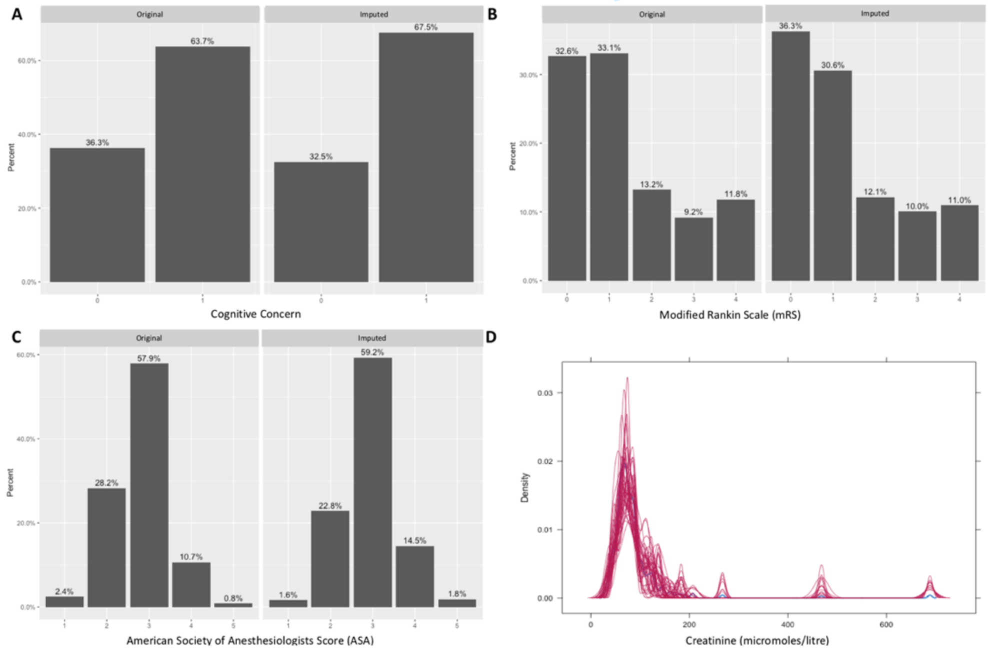

Supplementary Figure S4: *Visualisation of imputed values for A: Cognitive impairment documented on admission, B: Modified Rankin scale, C: American Society of Anesthesiologists (ASA) score, D: Creatinine.*

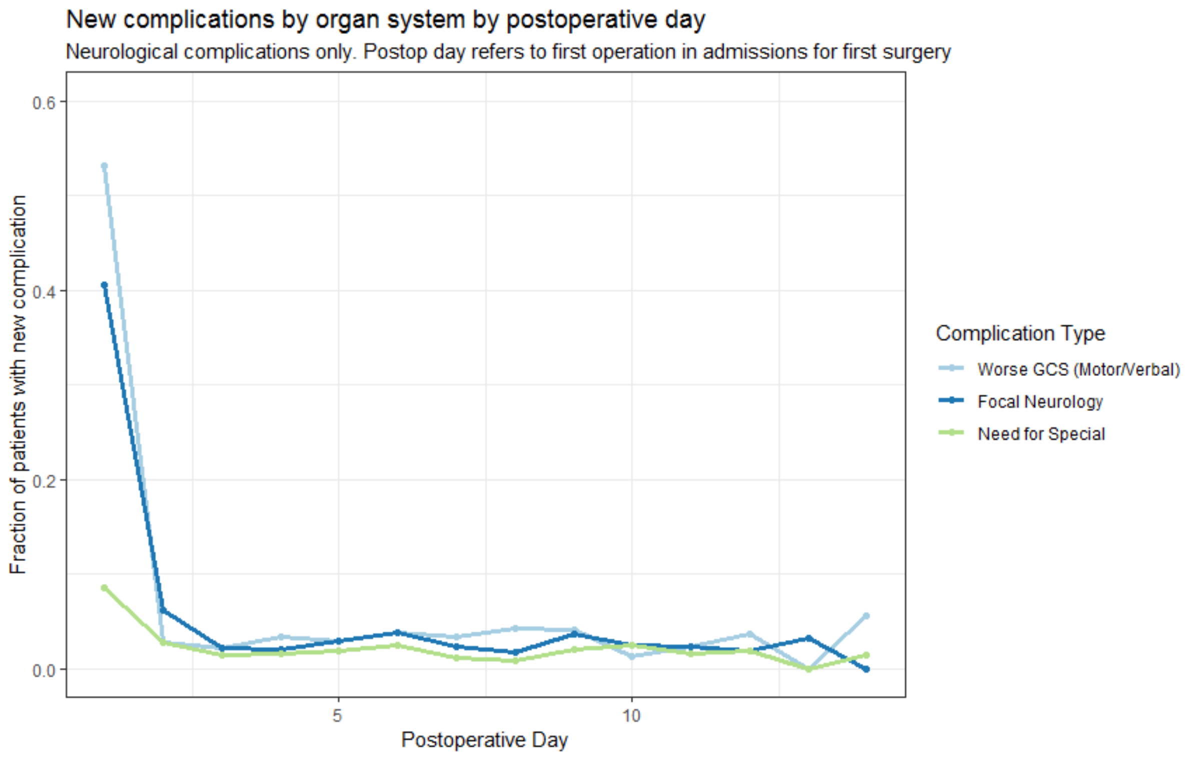

Supplementary Figure S5: Incident neurological complications by postoperative day.

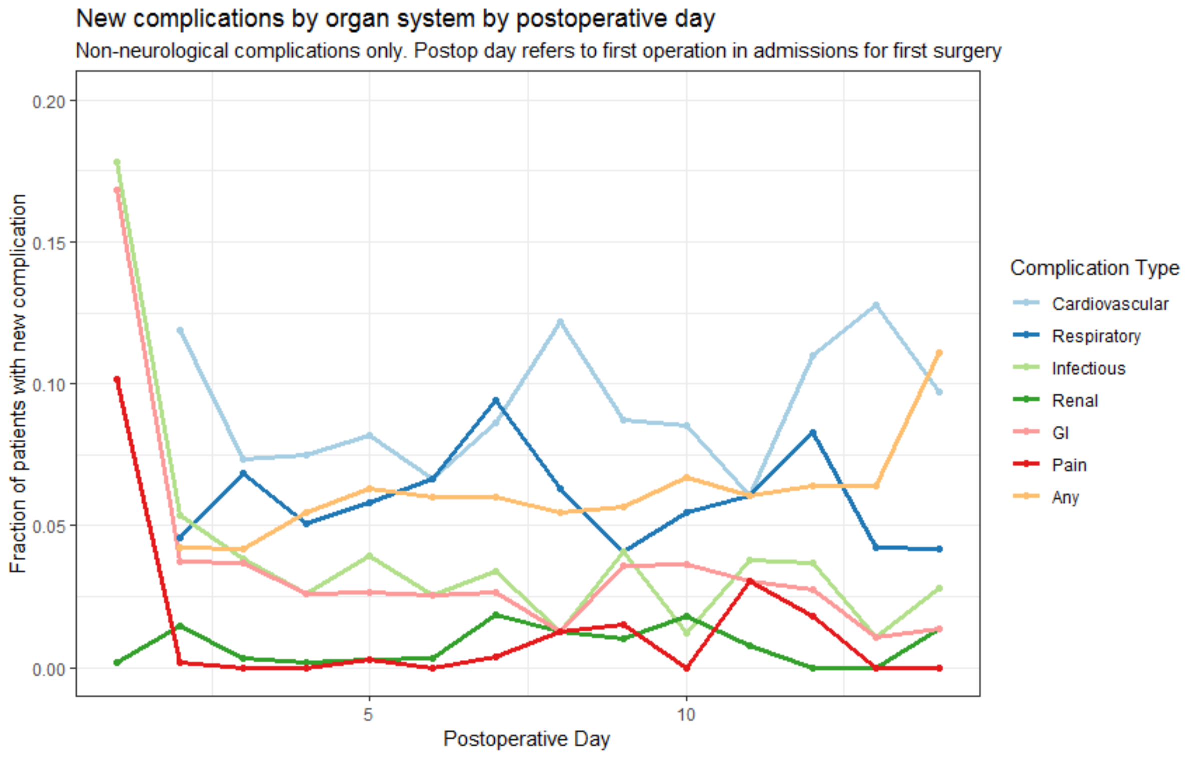

Supplementary Figure S6: Incident non-neurological complications by postoperative day.

	HR	2.5 %	97.5 %	*p*
**Demographics**				
**Age** *Per year*	0.993	0.987	1.000	0.042*
**Sex** *Male v Female*	1.204	0.998	1.453	0.053*
**Non-CUH patient**	2.197	1.541	3.131	<0.001*
**ASA 2** *All v ASA 1*	0.488	0.298	0.798	0.004*
**ASA 3**	0.338	0.208	0.547	<0.001*
**ASA4**	0.250	0.141	0.444	<0.001
**ASA5**	0.200	0.058	0.686	0.011*
**mRS 1** *All v mRS 0*	0.813	0.627	1.053	0.117
**mRS2**	0.837	0.593	1.182	0.312
**mRS3**	0.781	0.517	1.179	0.239
**mRS4**	0.670	0.456	0.985	0.041*
**Admission status**				
**GCS 15** *on admission versus any-other*	1.109	1.056	1.164	<0.001*
**Admission ePOMS** *per 1 domain increase*	0.915	0.865	0.967	0.002*
**Cognitive impairment** *Yes/No*	0.558	0.466	0.667	<0.001
**Comorbidities**				
**Airways disease** *Yes/No*	0.747	0.584	0.956	0.020*
**CVS disease** *Yes/No*	0.834	0.702	0.991	0.039*
**Heart Failure** *Yes/No*	0.698	0.560	0.870	0.001*
**Creatinine** *per umol/l increase*	0.958	0.922	0.995	0.028*
**Anticoagulated on admission** *Yes/No*	0.417	0.349	0.498	<0.001*
**Day of Surgery variables**				
**Pre-op deterioration** *(Yes/No)*	1.066	0.890	1.276	0.488
**Length of wait** *Per hour*	0.963	0.917	1.013	0.142
**Time MAP <80mmHg** *per 1 minute*	0.996	0.973	1.020	0.720
**Time *ETCO2* not 3-5kPa** *per 1 minute*	0.982	0.916	1.053	0.616
**Fentanyl dose** *per microgram increase*	1.012	0.988	1.037	0.330
**Volatile maintenance** *Yes/No*	0.996	0.838	1.183	0.964
**Operative time** *per 1 minute*	0.971	0.951	0.990	0.004*
**Postoperative**				
**Complications***per 1 domain increase in ePOMS*	0.574	0.521	0.632	<0.001*

Complete Case Analyses: Univariable analysis

Supplementary Table S2: Results from univariable cox regression analysis performed in complete cases for time to postoperative discharge after surgery for chronic subdural haematoma. *Hazard ratios (HR) of greater than 1 indicate an association with more rapid postoperative discharge. Asterisks indicate statistical significance at a p<0.1, threshold chosen for progression to multivariable modelling. Postoperative complications included as a time dependent covariate (by day). 95% CI = 95% Confidence interval. CUH = ASA = American Society of Anesthesiologists score, CUH = Cambridge University Hospitals NHS Foundation Trust, CVS = cardiovascular, ePOMS = electronic postoperative morbidity score, $ETCO_2$ = End tidal carbon dioxide measurement, GCS = Glasgow coma scale, MAP = mean arterial pressure, mmHg = millimetres of mercury, mcg = micrograms, mRS = modified Rankin scale.*

For peer review only

Complete Case Analysis: Multivariable analysis

	HR	95% CI	*p*-value
**Age** *(per year increase)*	0.99	0.98-1.01	0.301
**Sex** *(Male v Female)*	1.02	0.78-1.33	0.902
**GCS 15** *(v any-other value)*	1.03	0.93-1.15	0.581
**Referred patient**	2.40	0.69-8.30	0.167
**ASA** *(per 1 level increase)*	0.85	0.67-1.09	0.196
**Admission complications**	1.14	1.04-1.26	0.007*
**Airways disease** *(Yes/No)*	1.21	0.84-1.76	0.301
**CVS disease** *(Yes/No)*	1.13	0.84-1.52	0.424
**Anticoagulated on admission** *(Yes/No)*	0.52	0.40-0.69	<0.001*
**Heart failure** *(Yes/No)*	1.17	0.80-1.70	0.418
**mRS** *(per 1 level increase)*	1.03	0.93-1.13	0.566
**Baseline Creatinine** *(per 20umol/l increase)*	0.96	0.91-1.02	0.187
**Cognitive Concern** *(Yes/No)*	0.63	0.48-0.85	0.002*
**Operative time** *(per 10 minute increase)*	0.98	0.94-1.02	0.271
**Postoperative complications** *(per 1 domain increase in ePOMS)*	0.63	0.55-0.73	<0.001*

Supplementary Table S3: Results from multivariable cox regression model performed in complete cases (n = 260) for time to postoperative discharge after surgery for chronic subdural haematoma. *Hazard ratios (HR) of greater than 1 indicate association with a more rapid time to discharge. Asterisks indicate statistical significance at the 5% level. Postoperative complications included as a time dependent covariate (by day). 95% CI = 95% Confidence interval. CUH = ASA = American Society of Anesthesiologists score, CUH = Cambridge University Hospitals NHS Foundation Trust, CVS = cardiovascular, ePOMS = electronic postoperative morbidity score, GCS = Glasgow coma scale, mRS = modified Rankin scale.*

For peer review only

Sensitivity Analyses

	HR	95% CI	*p*-value
**Age** *(per year increase)*	0.99	0.99-1.00	0.169
**Sex** *(Male v Female)*	1.10	0.90-1.33	0.354
**Motor Score 6** *(v any-other value)*	0.97	0.83-1.12	0.674
**Referred patient**	1.53	1.01-2.29	0.042*
**ASA** *(per 1 level increase)*	0.88	0.75-1.04	0.135
**Admission complications**	1.05	0.99-1.12	0.102
**Airways disease** *(Yes/No)*	1.03	0.79-1.33	0.845
**CVS disease** *(Yes/No)*	0.96	0.78-1.19	0.715
**Anticoagulated on admission** *(Yes/No)*	0.45	0.37-0.54	<0.001*
**Heart failure** *(Yes/No)*	1.01	0.77-1.33	0.957
**mRS** *(per 1 level increase)*	0.99	0.91-1.08	0.835
**Baseline Creatinine** *(per 20umol/l increase)*	0.98	0.95-1.02	0.330
**Cognitive Concern** *(Yes/No)*	0.69	0.57-0.84	<0.001*
**Operative time** *(per 10 minute increase)*	0.99	0.97-1.01	0.374
**Postoperative complications** *(per 1 domain increase in ePOMS)*	0.61	0.55-0.68	<0.001*

Supplementary Table S4: Sensitivity analysis with total GCS replaced with an indicator variable for GCS motor score (6 or not) – highlighted in red.

For peer review only

	HR	95% CI	*p*-value
**Age** *(per year increase)*	0.99	0.99-1.00	0.046*
**Sex** *(Male v Female)*	1.15	0.94-1.39	0.168
**GCS 15** *(v any-other value)*	1.02	0.97-1.08	0.449
**Referred patient**	1.65	1.11-2.46	0.013*
**ASA** *(per 1 level increase)*	0.83	0.71-0.98	0.025*
**Admission complications**	1.01	0.95-1.07	0.841
**Airways disease** *(Yes/No)*	0.99	0.76-1.28	0.911
**CVS disease** *(Yes/No)*	0.96	0.78-1.19	0.732
**Anticoagulated on admission** *(Yes/No)*	0.45	0.37-0.54	<0.001*
**Heart failure** *(Yes/No)*	0.95	0.72-1.25	0.713
**mRS** *(per 1 level increase)*	0.97	0.89-1.07	0.561
**Baseline Creatinine** *(per 20umol/l increase)*	0.96	0.92-0.98	0.043*
**Cognitive Concern** *(Yes/No)*	0.65	0.53-0.79	<0.001*
**Operative time** *(per 10 minute increase)*	0.98	0.95-1.00	0.061

Supplementary Table S5: Sensitivity analysis, multivariable model with postoperative complication term excluded.

For peer review only

STROBE Statement—checklist of items that should be included in reports of observational studies

For: Utility of electronic health records in exploring associations between complex perioperative events morbidity and postoperative length of stay in surgically managed chronic subdural haematoma.
Stubbs et al.
(All page numbers refer to those in uploaded main word manuscript file)

	Item No.	Recommendation	Page No.	Relevant text from manuscript
**Title and abstract**	1	(*a*) Indicate the study's design with a commonly used term in the title or the abstract	2	
		(*b*) Provide in the abstract an informative and balanced summary of what was done and what was found	2	
**Introduction**				
Background/rationale	2	Explain the scientific background and rationale for the investigation being reported	3-4	
Objectives	3	State specific objectives, including any prespecified hypotheses	5	
**Methods**				
Study design	4	Present key elements of study design early in the paper	5	
Setting	5	Describe the setting, locations, and relevant dates, including periods of recruitment, exposure, follow-up, and data collection	5	
Participants	6	(*a*) *Cohort study*—Give the eligibility criteria, and the sources and methods of selection of participants. Describe methods of follow-up<br>*Case-control study*—Give the eligibility criteria, and the sources and methods of case ascertainment and control selection. Give the rationale for the choice of cases and controls<br>*Cross-sectional study*—Give the eligibility criteria, and the sources and methods of selection of participants	5	
		(*b*) *Cohort study*—For matched studies, give matching criteria and number of exposed and unexposed<br>*Case-control study*—For matched studies, give matching criteria and the number of controls per case		
Variables	7	Clearly define all outcomes, exposures, predictors, potential confounders, and effect modifiers.	6-8	

For peer review only - http://bmjopen.bmj.com/site/about/guidelines.xhtml

		Give diagnostic criteria, if applicable	
Data sources/ measurement	8*	For each variable of interest, give sources of data and details of methods of assessment (measurement). Describe comparability of assessment methods if there is more than one group	6-8
Bias	9	Describe any efforts to address potential sources of bias	Discussion
Study size	10	Explain how the study size was arrived at	5

Continued on next page

For peer review only

For peer review only - http://bmjopen.bmj.com/site/about/guidelines.xhtml

Quantitative variables	11	Explain how quantitative variables were handled in the analyses. If applicable, describe which groupings were chosen and why	8-9
Statistical methods	12	(*a*) Describe all statistical methods, including those used to control for confounding	8,
		(*b*) Describe any methods used to examine subgroups and interactions	
		(*c*) Explain how missing data were addressed	9 + Supplementary
		(*d*) *Cohort study*—If applicable, explain how loss to follow-up was addressed<br>*Case-control study*—If applicable, explain how matching of cases and controls was addressed<br>*Cross-sectional study*—If applicable, describe analytical methods taking account of sampling strategy	N/A
		(*e*) Describe any sensitivity analyses	11-12
**Results**			
Participants	13*	(a) Report numbers of individuals at each stage of study—eg numbers potentially eligible, examined for eligibility, confirmed eligible, included in the study, completing follow-up, and analysed	9 + Supplementary
		(b) Give reasons for non-participation at each stage	Supplementary
		(c) Consider use of a flow diagram	Supplementary
Descriptive data	14*	(a) Give characteristics of study participants (eg demographic, clinical, social) and information on exposures and potential confounders	10
		(b) Indicate number of participants with missing data for each variable of interest	9 + Supplementary
		(c) *Cohort study*—Summarise follow-up time (eg, average and total amount)	
Outcome data	15*	*Cohort study*—Report numbers of outcome events or summary measures over time	*9 + Fig 1*
		Case-control study—Report numbers in each exposure category, or summary measures of exposure	
		Cross-sectional study—Report numbers of outcome events or summary measures	
Main results	16	(*a*) Give unadjusted estimates and, if applicable, confounder-adjusted estimates and their precision (eg, 95% confidence interval). Make clear which confounders were adjusted for and why they were included	11-14
		(*b*) Report category boundaries when continuous variables were categorized	N/A
		(*c*) If relevant, consider translating estimates of relative risk into absolute risk for a meaningful time period	

For peer review only - http://bmjopen.bmj.com/site/about/guidelines.xhtml

For peer review only

Continued on next page

For peer review only

Other analyses	17	Report other analyses done—eg analyses of subgroups and interactions, and sensitivity analyses	11-12
**Discussion**			
Key results	18	Summarise key results with reference to study objectives	14
Limitations	19	Discuss limitations of the study, taking into account sources of potential bias or imprecision. Discuss both direction and magnitude of any potential bias	14-16
Interpretation	20	Give a cautious overall interpretation of results considering objectives, limitations, multiplicity of analyses, results from similar studies, and other relevant evidence	17
Generalisability	21	Discuss the generalisability (external validity) of the study results	17
**Other information**			
Funding	22	Give the source of funding and the role of the funders for the present study and, if applicable, for the original study on which the present article is based	18

*Give information separately for cases and controls in case-control studies and, if applicable, for exposed and unexposed groups in cohort and cross-sectional studies.

Note: An Explanation and Elaboration article discusses each checklist item and gives methodological background and published examples of transparent reporting. The STROBE checklist is best used in conjunction with this article (freely available on the Web sites of PLoS Medicine at http://www.plosmedicine.org/, Annals of Internal Medicine at http://www.annals.org/, and Epidemiology at http://www.epidem.com/). Information on the STROBE Initiative is available at www.strobe-statement.org.

For peer review only - http://bmjopen.bmj.com/site/about/guidelines.xhtml

For peer review only
